# Sustainable Career Transitions and Mental Health Support in Elite Sport: A Systematic Review of Evidence and Practices

**DOI:** 10.3390/sports13120438

**Published:** 2025-12-05

**Authors:** Francesca Di Rocco, Cristian Romagnoli, Simone Ciaccioni, Laura Capranica, Elvira Padua, Flavia Guidotti

**Affiliations:** 1 Department of Human Sciences and Promotion of Quality of Life, “San Raffaele” Open University of Rome, 00166 Rome, Italy; francesca.dirocco@uniroma5.it (F.D.R.); cristian.romagnoli@uniroma5.it (C.R.); elvira.padua@uniroma5.it (E.P.); 2 Department of Education and Sport Sciences, Pegaso University, 80013 Naples, Italy; simone.ciaccioni@unipegaso.it; 3 Department of Movement, Human and Health Sciences, University of Rome “Foro Italico”, 00135 Rome, Italy; laura.capranica@uniroma4.it

**Keywords:** sustainable career transitions, athletic identity, transition planning, mental health support, retirement adaptation

## Abstract

The present systematic review aims to provide a comprehensive synthesis of evidence and practices regarding sustainable career transitions in elite sport. Following PRISMA guidelines, an extensive literature search was conducted in SPORTDiscus (EBSCOhost), PsycINFO, Scopus, Web of Science, and Google Scholar databases, resulting in 117 manuscripts, published from January 2015 to May 2025, and meeting the defined inclusion criteria. The review focused on mental health, dual-career pathways, transition readiness, and identity-related issues among elite athletes, Olympians, and Paralympians. Methodologies included qualitative, quantitative, and mixed-methods designs, with multisport and mixed-gender samples prevailing. The most commonly used instruments were semi-structured interviews and surveys. The main findings highlighted the centrality of mental health support, the role of dual-career planning, and the importance of proactive identity negotiation. Despite growing research interest, significant gaps persist in access to psychological support, structured transition planning, and dual-career strategies, with notable inconsistencies across countries and sports. The review emphasizes the necessity for integrated, multidimensional guidance, culturally sensitive psychological services, and flexible educational pathways to promote athlete well-being and sustainable post-sport careers. These insights are intended to inform the implementation of the ERASMUS+ funded PORTAL project, supporting evidence-based interventions and the development of resources such as an online platform and Real-Life Transition Officers to enhance the transition experiences of elite athletes.

## 1. Introduction

Sustainability in sport presents both substantial challenges and valuable opportunities [1]. The promotion of quality education, lifelong learning, health and well-being, and access to employment in and through sport are key pillars for achieving sustainable development strategic goals [2]. Within this framework, the transition from elite sport to post-athletic life represents one of the most complex and challenging stages of an athlete’s career, often undermining the sustainability of long-term athlete support across different career stages. In particular, elite sport retirement is a multifactorial, longitudinal process rather than a singular event, characterized by profound psychological, social, financial, and vocational changes [3,4,5,6,7,8,9,10,11,12,13,14,15], involving multiple adjustments (e.g., identity reconstruction, skill transfer, reintegration into society, transition to the labor market), and highlighting the need for appropriate coping strategies and strategic career planning [16,17]. When not adequately managed, this transition can lead to a series of adverse consequences, including mental health disorders, financial insecurity, unemployment, and social isolation, which can negatively influence the well-being of former top-performance athletes and the sustainability of elite sport systems. These challenges highlight the importance of creating structured, comprehensive support mechanisms that promote long-term athlete welfare as an integral component of sustainable sport development [18,19].

One recurrent theme across the literature is the central role of athletic identity and its influence on post-retirement adaptation. Athletes with a highly exclusive sport identity, those who define themselves primarily through their athletic role, are at higher risk of maladaptive adjustments during retirement from sport, particularly in cases of forced or sudden retirement [17]. They frequently experience identity loss, decreased self-esteem, and difficulties envisioning a life beyond competitive sport [3,20]. In this regard, evidence suggests that gradual and planned transitions, combined with dual-career strategies, mental health support, and identity diversification are among the most effective protective factors against such negative outcomes [4,16].

Regarding the combination of elite sport demands and academic/vocational training (e.g., dual career), dual career pathways provide athletes with alternative career options by fostering an identity beyond sports, reducing the psychological risks associated with retirement [21]. Indeed, athletes who prepare for post-sport careers during their active years tend to experience smoother transitions, with the pursuit of education or vocational training alongside elite sports developing stronger social networks and professional connections, mitigating the emotional strain of leaving competitive sports [22]. Furthermore, integrating structured education and career development programs into athletic training can significantly reduce post-retirement anxiety, depression, and financial stress [23]. By encouraging educational engagement and professional development alongside athletic performance, these strategies foster resilience and facilitate integration into alternative roles, thereby contributing to the sustainability of long-term individual well-being [24,25]. In this regard, structured career transition programs and support services should be considered among key priorities and a responsibility for sport bodies at different levels (e.g., regional, national, international) [26], recognizing the need to safeguard elite athletes and their social capital as transferable assets in various sectors of the society at large [24,25].

Regarding psychological wellbeing, elite athletes are particularly vulnerable to psychological distress during career termination, often struggling to find meaning beyond their sport, potentially exacerbating mental health concerns. In particular, mental health symptoms rates among elite athletes have been reported as comparable to, and in some contexts higher than, those observed in non-elite athlete populations, with related evidence in student-athletes showing similar or elevated levels of distress compared with non-athlete students [19,27]. These vulnerabilities are often strengthened by abrupt or unplanned retirements due to injuries or deselection [3,5,16]. To note, responding to this growing concern, the International Olympic Committee (IOC) issued a Consensus Statement on Mental Health in Elite Athletes [28] and more recently launched its Mental Health Action Plan [29], both emphasizing the integration of mental health protection into organizational cultures rather than relying solely on crisis intervention [28,30,31]. These initiatives call for multi-dimensional strategies to normalize help-seeking behaviors, enhance mental health literacy among elite athletes and sport staff, provide early screening, and ensure adequate access to professional psychological services across all stages of an athlete’s career.

Comprehensive support systems are essential, given the profound psychological and emotional challenges associated with career transitions. To equip athletes with the skills and resources necessary to successfully transition in the post-sport life, the European Commission’s guidelines on dual careers [32] emphasized the importance of the provision of integrated services such as academic and athletic support, career counselling, and psychological support. In particular, psychological support tailored to the athletes’ needs (e.g., counselling, peer support, and mental health programs) plays a critical role in elite sports settings [21]. However, many athletes hesitate to seek mental health assistance due to stigma within the sports world [33]. For these reasons, making these services discreet, accessible, and culturally appropriate is crucial [23]. Similarly, career transition services including career counselling and job placement assistance (e.g., resume building, professional mentorship, vocational training) are likely to facilitate athletes achieving post-sport job opportunities, satisfaction, and financial stability [23]. To note, financial instability might become a pressing concern for athletes lacking financial literacy or post-retirement income plans [34]. Whilst elite athletes in major sports disciplines may earn substantial salaries, many others struggle with long-term financial security [23]. Social withdrawal and isolation are another concern, particularly for athletes who retire from highly structured team environments, with experiences of loss of peer support, feelings of loneliness and disconnection that might negatively influence the delicate stage of career retirement [17]. Thus, building strong social networks outside of sports through mentorship programs, peer support groups, and professional networking can provide emotional and practical support during career transitions [11,22].

In this framework, research and policy efforts should focus on enhancing tailored support systems, fostering stronger, long-term, impactful, and sustainable collaboration between different actors and stakeholders (e.g., sports organizations, academic institutions, counsellors, mental health practitioners) involved in athletes’ career assistance to ensure success in life, beyond sports competition [32]. Despite the recent policy advances in athletes’ welfare, significant gaps are still present in the availability and quality of support services, underscoring a persistent policy-practice gap and initiatives remaining mostly ad hoc and temporary rather than systemic and sustainable in the long-term [35]. In the context of elite sport, situating athletic retirement within a sustainability paradigm to go beyond individual coping strategies and encompass systemic equity, organizational accountability and resource durability represent a crucial aspect to minimize elite athletes’ mental, social, and financial burdens during post-sport life structural change and adjustment. According to the European Parliament [36], institutions should protect and promote the fundamental rights of athletes, including social rights, decent working conditions, occupational health, and safety principles. This should translate into efforts to ensuring that sport career termination is not a source of disadvantage but an opportunity for personal growth and reintegration [17,37]. Therefore, sustainable transition frameworks should embed mental health services, educational pathways, vocational training, career counselling, and financial planning throughout the athlete’s career, rather than concentrating efforts at the point of retirement. Such an approach supports several Sustainable Development Goals [2], reinforcing the relevance of sport as a vehicle for broader social sustainability.

Evidence from recent studies [3,5,16,17,19] highlighted the need of holistic, sustainable frameworks to facilitate the creation of athlete-centered systems that are scalable, inclusive, and integrated across sectors, transforming career termination from a vulnerability into a structured, empowering process. However, the achievement of these objectives requires multi-level governance engaging different actors and stakeholders (e.g., sport bodies, coaches and trainers, athlete commissions, educational institutions, healthcare systems, mental health practitioners, counselors, and policy-makers) in collaborative and coordinated actions. Furthermore, the implementation of international recommendations (such as the minimum standards on dual careers [38] and/or consensus/position stands on athletes’ mental and career support [7,39,40,41], remains fragmented at both national and local level. The translation of evidence-based knowledge into practice also lacks a systematic and coordinated approach. In particular, international recommendations are typically adapted variably across national contexts and translated into country-specific strategies. Due to differences in general and sport-specific policy frameworks, as well as different national athletes’ career assistance models [42], national action plans play a pivotal role in achieving meaningful outcomes. Consequently, governmental and sport institutions are tasked with developing targeted policies, funding initiatives, and implementing practical interventions to support elite athletes. By institutionalizing sustainable career support systems, sport organizations and policy-makers can ensure that elite athletes receive adequate resources and opportunities to thrive beyond their competitive years, safeguarding the ethical and social foundations of sport in alignment with long-term sustainability principles. However, these initiatives strongly rely on the availability and allocation of financial resources, which often undermine adequate support offered to elite athletes, and comprehensive, flexible, and sustainable support systems remain limited and uneven across countries [5]. In this respect, online digital resources beyond national borders could represent a tremendous opportunity to provide elite athletes with tools and concrete support to successfully cope with career retirement difficulties. In fact, the development of online digital resources such as e-counseling platforms, mobile applications, and career transition portals might offer a scalable and cost-effective strategy to bridge service gaps and deliver tailored support to athletes regardless of location or competitive status [43].

Digital tools can facilitate mental health screening, provide educational and vocational guidance, promote mental health literacy, and foster peer support communities, thereby addressing the multifaceted nature of sport career termination [44]. Policy initiatives and funding schemes at European level have begun advocating for the integration of digital innovation in athlete support frameworks, emphasizing accessibility, lifelong learning, and inclusive welfare provision. Indeed, embedding digital platforms into national and transnational support systems could enhance sustainability, equity, and long-term wellbeing for retiring elite athletes. To fill in this gap, the “Supporting Olympians Transitioning to Real Life in Concern of their Mental Health” (PORTAL) project has been recently funded by the ERASMUS+ Sport Programme of the European Commission to address the intricate challenges faced by elite athletes during their transition to post-retirement life. The project’s core objective is to develop and implement an inclusive online platform, complemented by a network of Real-Life Transition Officers, to provide comprehensive support to Olympians, particularly those facing fewer opportunities. To achieve these goals, a comprehensive knowledge of evidence- and eminence-based information is needed to equip the online platform with priority contents, tools, educational resources, access to employment and skill development opportunities, dissemination of open calls and events, a peer support community, and services specifically targeting career transitions, real-life, and mental health support for retiring high-performance athletes. Therefore, the present systematic literature review aims to synthesize contemporary scientific findings on career transitions, mental health, dual careers, and real-life support services for elite athletes within international sporting contexts. By identifying key trends, gaps, and evidence-based practices, this review will not only support the development of long-term, evidence-based strategies to elite athletes’ mental health and career assistance programs, but also to establish the creation of digital resources designed to operate sustainably beyond national borders and policy frameworks.

## 2. Materials and Methods

The present study was performed under the European ERASMUS+ Sport Lump Sum Grant “SupPorting OlympiAns Transitioning to Real Life in concern in their mental health—PORTAL” project (101184857-ERASMUS-SPORT-2024-SCP). The review was conducted following a predefined methodology approved by all partners through a consortium agreement and formalized in the project’s Methodological toolkit (Deliverable 1). The protocol was subsequently made publicly available through the International Prospective Register of Systematic Reviews (PROSPERO) to enhance transparency. No deviation occurred between the consortium-approved protocol and the procedures implemented during the review process.

### 2.1. Protocol Registration and Eligibility Criteria

The systematic literature review was retrospectively registered in PROSPERO (ID: CRD420251145490, available online: https://www.crd.york.ac.uk/PROSPERO/view/CRD420251145490, accessed on 1 September 2025) and based on the Preferred Reporting Items for Systematic Reviews and Meta-analyses-PRISMA guidelines [45] (Supplementary Materials File S1). To be included in the present review, selected studies had to meet the following criteria: (1) original peer-reviewed manuscripts in English language; (2) a time restriction set from January 2015 up to March 2025; (3) original articles without methodological restriction (i.e., quantitative, qualitative, and mixed methods) and study design; (4) content relevance in relation to research objectives; (5) address mental health, career, and retirement challenges and needs of elite athletic populations [46] showing one of the following characteristics:

- top-performers and/or world class athletes/para-athletes participating in major international competitions;
- athletes/para-athletes that participated in Olympic or Paralympic Games;
- athletes/para-athletes that achieved medals, titles or records in international competitions during their career;
- athletes/para-athletes involved in their respective senior National Team;
- team sports athletes/para-athletes playing professionally in major national leagues.

Although retained for other research purposes, reviews, consensus statements and meta-analyses have not been considered in the present study. Furthermore, studies involving youth, sub-elite, or student-athlete populations have not been considered in the present review.

### 2.2. Literature Search Process

In February 2025, a systematic literature search of original articles was performed on the following databases: SPORTDiscus (EBSCOhost), PsycINFO, Scopus, Web of Science, and Google Scholar. The following search string was used: (high-performance athletes career transitions) OR (elite athletes career transitions) OR (Olympic athletes career transitions) AND (high-performance athletes’ mental health) OR (elite athletes’ mental health) OR (Olympic athletes’ mental health) AND (high-performance athletes’ psychological health) OR (elite athletes’ psychological health) OR (Olympic athletes’ psychological health) AND (high-performance athletes’ support services) OR (elite athletes support services) OR (Olympic athletes support services). The asterisk (*) was used to pull all derivations of the similar root word (i.e., service/services). A detailed description of database-specific search strategies used for each database was reported in Supplementary Materials File S2. Google Scholar was used as a complementary source to identify records not indexed in the other databases. Search results were screened by title directly online, and all potentially relevant articles were saved in a predefined personal library for further screening (e.g., removal of duplicates, abstract screening and full-text download). To manage overlapping records, duplication was conducted in Excel (version 2017, Microsoft Corp., Redmond, WA, USA) by matching key bibliographic fields (i.e., title, first author, year, and DOI) and manually verifying duplicate entries. The literature search was performed on papers published from January 2015 to March 2025. To ensure the inclusion of the most updated articles in the present review, alert notifications of new publications were activated in all the included databases until the end of data collection (i.e., May 2025).

### 2.3. Study Selection and Data Collection Process

Three authors (F.D.R., F.G., and C.R.) specialized in Sport Science education, experts in dual career pathways of elite athletes, and involved in the PORTAL project independently screened the articles basing on title, keywords, abstract, and full text, reporting reasons for exclusion, ensuring compliance with defined inclusion criteria and pertinence in relation to the purposes of the present study. Specifically, the final eligible articles were equally distributed among the three primary authors, with each article independently assessed by two authors. When disagreement occurred between these two authors, a third author’s opinion was sought. A fourth specialized author (S.C.) was involved only when a disagreement on eligibility persisted after third evaluation.

### 2.4. Data Extraction

Three reviewers extracted the data of the final study sample. From the included articles, the following information was extrapolated and examined (Supplementary Materials File S3):

- bibliographic information (author(s), publication year, journal);
- geographical representation (e.g., country in which the study was conducted);
- general contextual information (e.g., dual career, mental health, career transition, support services);
- specific methodological information (e.g., sample, sample size, gender of participants, level of experience of participants (i.e., elite athletes, Olympians, Paralympic athletes) or data sources, type of study, dependent variables, applied instruments/tools)
- main outcomes specifically in relation to the objectives of the present study.

For both data extraction and study quality assessment, each included article was evaluated separately by three researchers, and in case of disagreement, a fourth author’s opinion was sought. Although the synthesis process followed a structured and transparent approach, the Synthesis Without Meta-Analysis (SWiM) approach was not applied [47], as the included studies were mainly descriptive, qualitative, or cross-sectional and did not provide quantitative effect estimates suitable for SWiM aggregation. Instead, data were synthetized narratively using a predefined extraction grid and explicitly grouping and classification rules to ensure methodological rigor and reproducibility, consistent with the underlying principles for the SWiM framework. Considering that no meta-analysis and/or sensitivity analysis was performed, certainty of evidence was judged qualitatively at the conclusion level (high/moderate/low/very low) using narrative criteria.

### 2.5. Quality Assessment

The Mixed Methods Appraisal Tool (MMAT)—Version 2018 [48], designed for the appraisal stage of systematic mixed studies reviews, was used to assess the methodological quality of each selected article. The MMAT allows the quality assessment of five categories of studies: qualitative research, randomized controlled trials, non-randomized studies, qualitative descriptive studies, and mixed-methods studies. The appraisal includes two preliminary screening questions: (1) Are there clear research questions? and (2) Do the collected data allow to address the research questions? followed by five specific questions according to the study typology. Each question requires a rating through a positive (e.g., “Yes”), negative (e.g., “No”) or doubtful (e.g., “Can’t tell”, applicable when appropriate information to answer “Yes” or “No” are not present and/or unclear) answer. Whether the answer is “No” or “Can’t tell” to one or both the screening questions, further appraisal may not be feasible or appropriate. In addition, a cross-study risk-of-bias synthesis was conducted by aggregating item-level judgments (“Yes”, “No”, “Can’t tell”) for each of the MMAT checklist items. The resulting summary table and 100% stacked horizontal bar plot are presented in Supplementary Materials File S4.

## 3. Results

### 3.1. General Findings from the Systematic Literature Review

**Figure 1:**
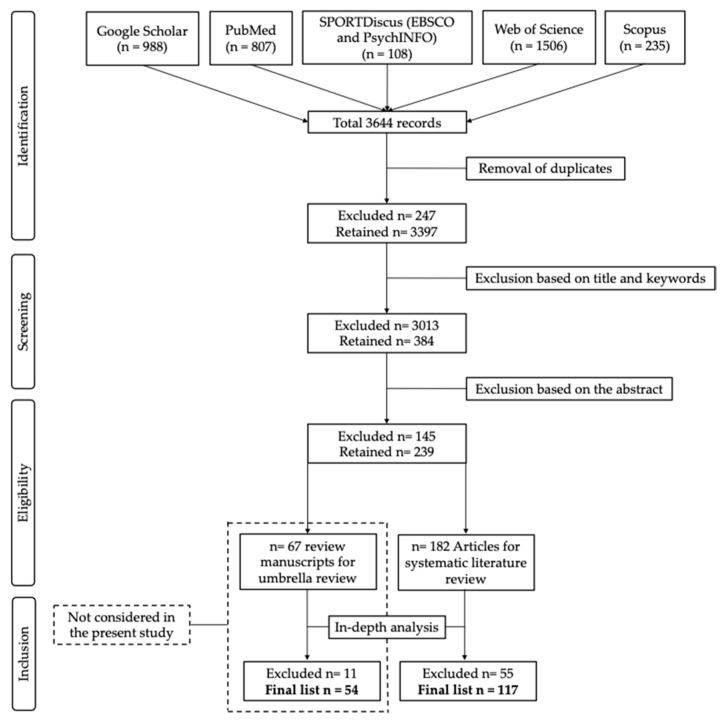
Flowchart representing the systematic process of review of the present study.

The initial search across five databases (i.e., EBSCOhost, PsycINFO, Scopus, Web of Science, and Google Scholar) yielded 3644 records. After the removal of duplicates, title/keywords screening, and abstract evaluation, 239 papers were retained for in-depth analysis. Among them, 67 review manuscripts were not considered in the present study, whereas 182 primary studies were processed for data extraction. Fifty-five studies have been excluded because they were outside the scope of the present systematic review. One-hundred-seventeen manuscripts met the inclusion criteria (Figure 1) and have been included in the final list, as presented in Table 1.

The retained manuscripts were published in a range of leading scientific journals, such as Psychology of Sport and Exercise (*n* = 16 studies), Frontiers in Psychology (*n* = 9 studies), Journal of Applied Sport Psychology (*n* = 7), Journal of Clinical Sport Psychology (*n* = 6), and BMJ Open Sport & Exercise Medicine (*n* = 5).

For geographical representation, the included studies resulted in the highest occurrence for Europe (*n* = 68, 57.50%, with the United Kingdom as the most represented country), followed by North America (*n* = 20, 17.09%, with Canada as the most represented country) and Oceania (*n* = 11, 9.40%, with Australia as the most represented country). Furthermore, eight studies included countries from different continents [50,55,66,94,110,111,139,159]. Lower frequencies of occurrence emerged for Asia (*n* = 7, with China as the most represented country), Africa (*n* = 2), and South America (*n* = 1).

Regarding the general research focus, the majority of the studies addressed mental health support to elite athletes (47.01%) [4,49,52,55,56,58,63,64,65,70,78,79,80,81,82,84,85,86,89,94,95,97,102,104,105,106,109,111,112,115,117,121,122,124,126,129,136,137,138,141,143,146,149,150,151,152,154,155,156,157,158,159,160,161], followed by career transition out of sport and retirement (40.17%) [18,44,50,51,53,54,55,57,59,60,62,66,67,68,69,71,73,74,75,77,83,88,90,91,92,93,96,98,99,100,101,103,113,114,116,120,123,127,130,132,135,139,140,142,147,148,153], whereas career support (7.69%) [42,50,61,87,107,108,118,119,125,131] and athletic identity and retirement (5.13%) [72,76,110,128,144,145] were less frequently represented.

### 3.2. Characteristics of the Included Studies

#### 3.2.1. Participants’ Characteristics, Methodologies, Instruments, and Quality Assessment of the Included Studies

The included studies presented both qualitative (*n* = 65, 55.56%) [4,42,44,49,50,51,53,54,55,57,58,59,60,63,65,66,67,68,70,72,74,75,76,78,81,84,92,93,98,100,101,102,103,105,106,107,108,109,110,113,114,115,118,119,120,123,124,125,126,127,129,130,132,135,139,143,144,147,148,151,152,153,155,157,159,161], and quantitative (*n* = 44, 37.61%) [18,52,56,61,62,64,77,79,80,82,83,85,87,88,90,91,94,95,96,97,104,111,112,113,116,117,121,122,126,128,131,133,134,136,137,140,141,142,145,146,149,150,154,158,160] approaches. Additionally, mixed methods (i.e., qualitative and quantitative) emerged in eight studies [69,71,73,86,89,99,138,156]. Qualitative research included in the present study relied mostly on semi-structured or unstructured interviews, frequently analyzed through interpretative approaches such as reflexive thematic analysis or Interpretative Phenomenological Analysis [44,51,54,55,61,65,66,68,70,72,75,76,78,79,81,84,86,93,100,101,102,103,107,108,109,114,124,125,127,130,132,139,143,144,147,148,152,153,155,157,159,160,161]. Several studies adopted innovative qualitative strategies, including autoethnographic vignettes, polyphonic reflective tales, or narrative/theoretical analysis [53,57,110,123,129]. Regarding quantitative instruments, the majority of the studies employed structured questionnaires and online surveys, either tailored or based on internationally validated instruments, such as the Mini-International Neuropsychiatric Interview [111], the Montgomery-Asberg Depression Rating Scale [83,141], and specific tools to assess psychological well-being, athletic identity, or coping resources [18,52,62,63,64,73,74,77,80,86,88,89,90,91,95,104,105,106,116,117,119,122,128,131,136,137,145,146,150,154,156,158]. Several works also integrated quantitative and qualitative methodologies into mixed methods approaches that combined surveys/questionnaires with interviews or focus groups [4,50,61,67,71,138,147]. Finally, some studies used specific instruments tailored to their target population, such as neurocognitive assessment batteries for retired rugby players [140], health questionnaires for elite athletes [112], or adapted scales for maltreatment [126].

Participants in the included studies represented a comprehensive range of key stakeholders within the elite sports environment. The majority of studies focused on elite athletes, encompassing Olympic athletes, Paralympic athletes, and national or professional competitors from diverse sporting backgrounds [4,18,44,49,50,51,52,53,54,55,56,57,58,59,60,61,62,64,65,66,67,68,69,70,71,72,73,75,76,77,78,80,81,82,83,84,85,86,87,88,89,90,91,93,94,95,96,97,98,99,100,101,102,103,104,106,107,108,109,110,111,112,113,114,115,116,117,118,119,122,123,124,125,126,127,128,129,130,131,132,133,134,135,137,138,139,141,142,143,144,145,146,147,148,149,150,151,152,154,155,156,158,159,160,161]. Several studies specifically examined retired high-performance athletes, thereby providing insights into the long-term effects of athletic career transitions and post-retirement adaptation [18,44,51,61,62,66,67,69,72,73,75,76,81,82,88,91,100,104,110,130,138,145,147,159]. In addition to athletes, some studies involved coaches, sport psychologists, and sport management staff, highlighting the perspectives of those responsible for athletes’ development and well-being [103,125,143,153,156,157]. The samples spanned over multiple sports disciplines, reflecting the diversity of the elite sporting landscape. Whilst a primary focus on those competing or having competed at the highest level (e.g., Olympic Games, Paralympic Games, world championships, professional leagues) emerged, a few studies included also sub-elite, youth, or club-level athletes [154], and some considered the perspectives of athletes undergoing the transition to post-sport careers, including transitions into coaching or other professions [103,120].

Overall, the included studies encompassed more than 30,000 participants, with individual sample sizes ranging from single case studies (*n* = 1) to large-scale surveys involving over 8000 respondents. The majority of studies reported sample sizes between 10 and 500 participants, reflecting substantial variability in study designs and scopes.

Regarding the participants’ gender, this information was not reported in four studies [83,89,103,118]. Most studies (*n* = 81, 69.23%) included both male and female participants (mixed-gender samples), whereas 23 studies (19.66%) included only male participants and 9 studies (7.69%) focused exclusively on female participants.

Regarding sport typology, most studies included samples from multiple sports (n = 81, 69.23%) [4,18,42,44,49,50,51,54,55,56,57,59,61,62,63,64,65,66,67,68,72,73,74,75,76,77,78,85,86,87,88,90,91,92,93,95,96,98,99,100,101,102,103,105,106,108,109,110,111,114,116,117,119,121,122,125,126,127,128,129,131,135,136,137,138,139,143,145,147,149,150,151,152,153,155,156,157,158,159,160,161], whereas 24 studies (20.51%) focused exclusively on team sports [52,60,69,70,71,79,80,81,82,89,94,96,97,104,112,113,115,123,133,134,137,140,142,146], and 10 studies (8.55%) involved individual sports only [53,58,84,107,124,130,132,141,144,148]. In 2 studies [83,118], the type of sport was not specified.

The evaluation of the quality of included studies is presented in Supplemental File S4. The diversity of research designs, populations, and methodologies reflects both the strength and challenge of the existing evidence. In fact, although comprehensive, the literature’s fragmentation complicates the identification and implementation of adaptable and sustainable tools for practice. Furthermore, the heterogeneity of included studies elicited a moderate certainty of evidence (84%) as presented in Supplemental File S3. In fact, cross-sectional designs exceeding those of longitudinal nature, sample sizes varying considerably among included studies, small or convenience samples, self-reported data collection approaches, limited validated tools/instruments, and cultural heterogeneity and variability in elite-sport structural systems across countries may constrain the generalizability and transferability of findings.

#### 3.2.2. Relevant Topics Emerged in the Present Systematic Literature Review

The recent literature on athletic retirement and transition evaluated in the present study underscores a broad and multifaceted set of themes, reflecting the complexity and diversity of athletes’ experiences both during and after their sporting careers. The included studies span a wide array of settings, methodologies, and participants groups, reinforcing the robust but fragmented nature of this research area (Supplementary Materials File S2).

One of the most salient emerging topics concerns the interconnection between athletic identity and mental health. Studies consistently showed that the phases of athletic identity development, crisis, and reconstruction are intimately tied to fluctuations in mental health, with particular vulnerability observed during moments of injury or abrupt career interruption [57,65,70,83,139,155]. The loss of athletic identity often coincides with increased symptoms of depression, anxiety, and psychological distress, and may lead to avoidance-based coping or even identity collapse for those athletes strongly identifying themself with their sport [57,70,146]. Conversely, processes of identity renegotiation and proactive support, such as interventions focused on fostering new roles, have been shown to facilitate more adaptive career transitions [139,144]. However, few interventions in the literature demonstrated scalability or sustainable impact beyond specific populations or contexts.

The role of cultural, gendered, and social context in shaping retirement experiences has also been extensively addressed in the literature. Included studies illustrate how retirement trajectories are affected by sociocultural variables, such as gender expectations, family and social support, and national sporting structures. For example, female athletes often face specific challenges related to health, social pressures, and the lack of support, while Indigenous and para-athletes experience unique barriers linked to identity, stereotypes, and social constraints [50,51,55,67,123]. These factors point to the need for accessible, adaptable, and culturally informed tools as an essential consideration for the development of digital resources to support elite athletes beyond national borders.

Another steady theme in the literature is the impact of career planning and dual-career management on post-retirement adjustments. Athletes who engage in structured dual-career pathways, combining sport with education or employment, report smoother transitions and greater well-being after retirement [50,87,88,96,116]. The presence of anticipatory career planning, as well as organizational support in skills development and mentoring has been consistently identified as a key protective factor [42,44,100,128]. These findings support the integration of modular, evidence-based tools for proactive planning within sustainable digital platforms.

The literature also foregrounds mental health challenges and risk factors associated with retirement. Across sports disciplines and countries, there is a relevant prevalence of symptoms such as depression, anxiety, eating disorders, and substance misuse among elite athletes, especially those more vulnerable, which in some worse cases might lead to suicidal ideation [94,95,111,115,122,146]. These symptoms are often exacerbated by factors such as injury history, cumulative stress, abrupt or involuntary retirement, lack of social support, and the stigma associated with help-seeking [52,56,70,89,97,132,138]. Hence, both informal (e.g., family, peers) and formal (e.g., psychologists, counsellors, educators in career transition programs) social support has been recognized as a means of buffering negative outcomes [60,107,130,138,143]. However, the implementation of support services into permanent, accessible, and scalable digital formats is rarely discussed in the current literature.

A further area of interest involves the psychosocial and structural resources currently available (or in development) for elite athletes, including life skills development, financial literacy, and career support services. Recent studies pointed out that resources such as mentoring, counselling, and post-career transition programs can enhance life satisfaction, employability, and positive psychological adjustments, although access remains uneven and often limited to medalists in major international competitions [42,44,77,90,100,118]. The need for broadly available, digitalized, and sustainable tools is therefore a clear implication for practice.

Finally, the ethical and organizational dimension of athletes’ career transition and retirement is also gaining scientific attention. Indeed, research has begun to question the roles and responsibilities of sports organizations, coaches, and policy-makers in facilitating healthy and sustainable career transitions and safeguarding elite athletes’ well-being [125,130,153,157]. This includes addressing not only the prevention of maltreatment, abuse, and psychological harm, but also the proactive creation of environments that promote holistic development and well-being beyond sport, with few existing systems only demonstrating effective mechanisms for ongoing evaluation, adaptation, or sustainability, which represent key features for future interventions [126,135,152,160].

Overall, the recent literature underlines the multifactorial nature of elite athletes’ career transitions, where personal, social, cultural, and organizational factors intersect to influence their post-sport life. In particular, a clear consensus emerged on the need for more individualized, culturally sensitive, and integrative approaches to support elite athletes during this critical life stage. However, the field still lacks a consolidated, practical roadmap for sustainable, long-term, and transnational support.

### 3.3. Outcomes Informing the Implementation of Support Digital Resources (Services and Best Practices)

The included studies offer a multidimensional body of evidence and recommended practices relevant to the development of sustainable, digital support resources for elite athletes’ career transition and mental health support. Main information and findings from included manuscripts are summarized in Table 2, with a particular emphasis on outcomes that might be relevant for the development of a sustainable, transnational online support digital resource (e.g., such as the PORTAL project platform). Overall, the in-depth screening of included manuscripts revealed a vast and well-established body of work addressing the psychological, social, and practical dimensions of athletes’ career transitions. However, few studies only provided direct guidance on the long-term, cross-national sustainability of support interventions, highlighting a significant gap that needs to be addressed through innovative support strategies.

A consistent theme is the necessity of a holistic and individualized support spanning mental health literacy, dual-career planning, post-retirement assistance, and ongoing psychological care. Many authors emphasized that effective frameworks must involve collaboration among all stakeholders (e.g., coaches, sport psychologists, counsellors, peers/family, sports organizations), who should address not only the athletes’ immediate performance needs but also their long-term development and well-being. However, few interventions have demonstrated true scalability or cross-cultural adaptability, reinforcing the need for integrated, transnational, and sustainable resources [58,65,109,124,125,138,155].

Relevant recommendations highlighted the promotion of identity diversification and resilience, both during and after the sporting career, envisaging interventions that support athletes in exploring alternative roles, maintaining connections outside sport, and engaging in proactive retirement planning [57,66,71,86,125,135,138,147,155]. Good practices also include the integration of psychological and educational resources within digital platforms, enabling athletes to access peer mentoring, mental health screening, and individualized learning spaces [71,120,135,161].

Mental health support is consistently highlighted as an urgent need. The evidence suggests that services should go beyond crisis intervention to include preventive education, stigma reduction (especially via digital communities and narratives), and easy access to psychological services both during high-pressure competitive phases and career retirement [50,65,66,86,135]. Notably, some studies recommend that mental health education and literacy should be introduced during the early stages of athletic development, normalizing help-seeking behaviors and creating psychologically safe environments [86,95].

From a service delivery perspective, digital and blended approaches are deemed valuable for their flexibility and potential to reach a broad population of athletes, including those who are retired or transitioning to new careers [50,71,135]. Effective practices should include online peer support forums, self-assessment tools, and digital mentoring schemes. The importance of monitoring and follow-up is also underlined, especially to identify athletes who may be at risk of declining mental health or presenting maladaptive adjustments to career retirement [71,86,135].

Cultural and gender-specific factors are frequently discussed. Good practices stress the need for culturally competent services and the recognition of unique needs among female, para-, and minority athletes [51,81,124,141]. Collaborative interventions that leverage the lived experience of retired athletes as mentors or role models are particularly effective in both career and mental health outcomes [71,73,86,135]. Effective, sustainable platforms must ensure inclusivity and adaptability to diverse cultural, gender, and ability-based needs. In this framework, a transnational digital platform could foster the integration of culturally competent resources and best practices, moving beyond sector- or context-specific limitations.

Finally, a few studies underline the necessity of clear role definitions and cooperation among all stakeholders, sports organizations, coaches, mental health professionals, and digital platform providers, to deliver comprehensive support systems spanning over the entire career life cycle [58,120,135]. Furthermore, ensuring the sustainability of athletes’ support interventions requires not only robust stakeholder collaboration but also the operationalization of clear guidelines, ongoing quality monitoring and implementation, and evidence-based evaluation tools.

Core tools and practices with potential for sustainable, cross-national implementation could be summarized as follows:

- digital self-assessment modules for mental health and career transition readiness;
- asynchronous peer mentoring networks;
- modular educational contents on dual-career management, career planning, financial literacy, and identity development;
- confidential, online access to professional support; and
- family/community engagement in athletes’ support programs.

## 4. Discussion

The present systematic review offers a comprehensive synthesis of recent scientific findings on career transitions, mental health, dual careers, and support systems for elite athletes, with a particular focus on sustainable, long-term services implementation though cross-national innovative digital tools. The body of evidence is extensive and well established, highlighting the multidimensional and complex nature of athletes’ post-career experiences, the diversity of risk and resilience factors, and the ongoing evolution of approaches to holistic athlete development. However, a lack of practical, sustainable, innovative, and transnational approaches and tools has been identified. Therefore, the development of research-informed digital support resources for elite athletes across diverse cultural settings is highly needed.

Despite the breadth and depth of the existing literature in this field, a meta-analysis was considered not feasible due to heterogeneity in study designs, methodologies, and outcome measures. However, the present review attempted to transparently evaluate the robustness of the evidence base. In particular, the methodological quality was appraised using the MMAT, allowing the evaluation of studies presenting different study designs and methodologies through a single evaluation instrument. To note, regarding the confidence of evidence, the majority of studies demonstrated moderate methodological rigor. In particular, qualitative studies were found to consistently employ systematic thematic or interpretative approaches. Furthermore, most of the quantitative studies adopted validated instruments and structured research protocols. Nevertheless, several recurrent limitations emerged, affecting the overall strength of inference. Firstly, several included studies adopted cross-sectional designs, limiting causal interpretation and longitudinal extrapolation. Secondly, sample sizes varied considerably among included studies, with several manuscripts relying on small or convenience samples, potentially constraining generalizability and transferability of findings. Thirdly, the analyzed literature showed self-reported data collection as the most represented approach, introducing potential risks of recall and social-desirability bias. Moreover, cultural heterogeneity and variability in elite-sport structural systems across countries may contribute to contextual differences that warrant careful interpretation when extrapolating findings across sport settings. Overall, these considerations support a moderate level of confidence in the qualitative and thematic evidence addressing the multidimensional nature of athletes’ career transition and assistance domains. In contrast, confidence in the generalizability of quantitative evidence is deemed low-to-moderate, due to limited longitudinal studies, variability in measurement tools, and non-probabilistic sampling in several studies. Future research adopting longitudinal and mixed methods designs, enhanced reporting standards, and cross-cultural comparative frameworks is needed to strengthen certainty and guide the development of sustainable and scalable support strategies for elite athletes.

Athletic identity has been consistently identified as a double-edged sword in career transitions. Whilst a strong, exclusive athletic identity may increase vulnerability to psychological distress during and after retirement, particularly in case of abrupt or involuntary career termination, the development of multiple and flexible identities is based on the proactive commitment of athletes, facilitating effective adaptive transition and psychological well-being [57,70,81,138,139,155]. Accordingly, interventions that foster identity diversification, self-reflection, and early planning are repeatedly recommended. However, translating these principles into accessible, scalable, and sustainable support tools remains a challenge that few studies address directly.

The prevalence of mental health symptoms among elite athletes, both active and retired, represented a strong concern in the literature, often matching or exceeding rates observed in the general population [52,56,64,86,94,95,106,111,117,146,149]. Risks are further heightened for female athletes, para-athletes, and those experiencing injury or abrupt retirement. Performance pressures, limited social support, cumulative stress, and stigma contribute to these challenges, as do systemic issues such as maltreatment and psychological neglect [52,86,94,97,126,132,135,138]. Thus, culturally informed interventions should be envisioned, ensuring equal access to mental and career assistance services to a broader elite athletes’ community, going beyond international sporting successes often guiding resources allocation and preventing the “left behind” pathway characterizing the most vulnerable individuals. Whilst many interventions emphasize prevention, early identification, and tailored support, only a minority address how such services should be delivered sustainably, across contexts, and over the athlete’s lifespan. This underscores the need for digital, regularly updated, and easily accessible tools.

Recognizing early warning signs, normalizing mental health within the diverse sports settings, and providing both preventive and therapeutic interventions are universally acknowledged as best practices [50,89,115,126,132]. Digital strategies, including peer-led storytelling, culturally competent education, confidential self-assessment tools, and regular mental health monitoring and personalized support throughout the athletic career and retirement emerged as promising means to reduce stigma and enhance engagement [129,132,135], although the long-term maintenance, scalability, and cross-cultural relevance of these strategies need to be addressed.

Dual career pathways, combining sport with academic or vocational pursuits, consistently protect against adverse mental health outcomes and support smoother post-sport transitions [18,44,50,54,87,88,95,116,128,136]. Athletes who engage in anticipatory planning, skills development, and mentoring enjoy greater well-being and post-retirement opportunities. Persistent disparities, however, reflect gaps in accessibility and equity, often determined by gender, sport type, socioeconomic status, and national context [55,62,87,91,162]. Digital platforms that incorporate modular, adaptable dual-career resources, as envisioned in recent European initiatives and projects [35,163]. In particular, the PORTAL project intends to offer a scalable solution to bridge these divides and sustain support beyond local or temporary initiatives.

Social support remains a vital moderator of both psychological well-being and successful career transitions [4,65,77,95,130,138,143]. Whilst athletes often favor informal, trusted networks, these relationships can be strained by career transitions and/or relocations [164], underscoring the importance of family-inclusive and community-based approaches [165]. Critically, the literature highlights the vulnerability athletes experience post-retirement, often citing a sense of abandonment and loss of support [42]. This signals a pressing need of maintaining proactivity and active engagement in career planning and support, and of delivering support consistently over time and across geographic boundaries.

Environmental factors, such as organizational culture, coaching style, and climate of care, profoundly shape athletes’ experiences [125,126,135,153]. Coaches and sports organizations may serve as either protective or risk factors, depending on the clarity of roles, educational efforts, and stakeholder accountability. Sustainable support structures must therefore embed continuous professional development, regular evaluation, and a culture of care within all levels of the sporting ecosystem.

Cultural, gender, and social contexts exert strong influences on the retirement and transition experiences of athletes. Distinct barriers, including discrimination and insufficient tailored support, have been reported to affect female athletes, Indigenous, para-athletes, and minority groups [51,55,62,67,123,124,126]. Addressing these challenges requires culturally sensitive, inclusive interventions and ongoing competence training. Furthermore, cross-national variability in policies and resources demonstrates the necessity for digital resources offering adaptable, evidence-based guidelines, supporting athletes irrespective of national boundaries, thus advancing the concept of sustainability “beyond borders”.

The long-term sustainability of athlete support systems strongly relies on robust stakeholder collaboration, including major sports organizations, educational institutions, healthcare providers, the labor market environment and policymakers [24,58,100,120,135]. Clear role definitions, regular follow-up, and continuous evaluation are vital to ensure services remain relevant and effective. Aligning with international sustainability frameworks (e.g., the UN Sustainable Development Goals and IOC consensus statements), these principles are integral to the PORTAL platform’s vision of lasting, adaptable, and system-wide support. Overall, effective athletes’ mental health and career transitions support systems must combine psychological, educational, and practical resources, delivered in accessible, personalized, and adaptable formats. Digital and blended platforms stand out for their ability to reach broad and diverse populations, overcoming geographical, temporal, and social barriers. Key tools, such as peer mentoring, mental health literacy modules, financial education, and career guidance, are consistently recommended as foundational to sustainable, impactful support systems. In this framework, the present study represents a milestone to inform the PORTAL project’s platform implementation, which would synthesize these priorities operationalizing the most robust evidence and best practices into a single, scalable, and transnational digital environment.

### Practical and Digital Implementation Implications

While the present review was not designed to directly test digital interventions, the evidence synthetized provides a conceptual foundation for informing the development of scalable, evidence-based resources such as the PORTAL platform. To note, findings suggest the strong need to bridge services gaps, enhance accessibility, and promote sustainability in athlete support systems. The integration of educational, psychological, and vocational modules, along with culturally sensitive and gender-responsive content, appears particularly relevant for improving reach and impact. Therefore, implementing digital tools might represent an innovative, user-friendly, up-to-date, flexible, and cross-national strategy to meet this gap. In fact, an online platform providing access worldwide and offering relevant and necessary support services to both active and retiring elite athletes could help them manage their post-sport career planning, mental health, and retirement transition. Such an approach could align with the principles of sustainable development (e.g., United nations Sustainable Development Goals: UN SDG 3, SDG 4, SDG 17), the IOC Consensus Statement on Mental Health in Elite Athletes (2021), and the Sustainable digital health interventions strategies [166]. In this framework, the PORTAL online platform could represent a valuable opportunity to translate scientific evidence pertaining elite athletes’ needs into a best practice through a digital support ecosystem, designed to operate beyond boarders and in the long term. However, future studies should investigate the effectiveness of implementing such digital and cross-national support approaches to meet the actual elite athletes’ needs. In fact, considering the variety of contextual determinants of career success and retirement transition (e.g., gender, type of sport, dual career pathways, sport environment, culture, community, facilities, resources, career development and planning tools), empirical validation of such digital approaches is needed in future research.

## 5. Conclusions

This systematic review confirms the complexity and multidimensionality of elite athletes’ career transitions and mental health challenges, highlighting the urgent need for integrated, proactive, and individualized support across the athletes’ lifecycle. Early, holistic preparation, especially through dual career pathways, psychological support, and anticipatory career planning remains critical for building resilience, personal development, well-being, and employability opportunities beyond sport. Persistent barriers, such as stigma, organizational disparities, and the specific vulnerabilities call for innovative and inclusive solutions.

Notably, our review identifies a clear gap between the richness of the academic literature and the development of practical, sustainable, and transnational tools. Although several studies highlighted the importance of psychological assistance during and after athletic retirement, only a limited number provided quantitative data on how many athletes effectively received such support. Most qualitative evidence revealed that psychological services for elite and retired athletes remain fragmented, inconsistently available, and often stigmatized, with many athletes reporting unmet needs for mental health counselling or transition guidance. This lack of structured data and service provision exemplifies the existing gap between the extensive scientific evidence on mental health in sport and its limited translation into sustainable, transnational support frameworks.

Coordinated stakeholder efforts, digital and blended platforms, family-inclusive methods, and culturally tailored interventions all emerge as essential strategies to promote sustainability and real-world impact. Future programs should adopt a tailored and context-sensitive approach, considering the unique needs of elite athletes and high-performance athletes across sporting disciplines, gender and stages of the career transition. Ongoing evaluation and policy alignment are vital to ensure that best practices are continually refined and adapted to the evolving realities of sport. By embracing a holistic, athlete-centered, and sustainability-driven approach, stakeholders can contribute to more positive and enduring transitions for elite athletes, empowering them to succeed not only in sport but in life after competition, and ensuring that support remains available “beyond borders”. Within this framework, the insights derived from this systematic literature review may inform the development of evidence-based, digital, and sustainable resources, such as the ongoing PORTAL project aimed at strengthening long-term support systems for elite athletes.

This systematic review presents some limitations that should be acknowledged. Firstly, although multiple databases were systematically searched and updated alerts were used to ensure inclusion of the most recent publications, relevant studies may have been missed, particularly given the growing research interest towards athletes’ career and retirement assistance. Secondly, the included studies were heterogeneous in terms of methodology, design, and outcome measures, limiting the ability to conduct a meta-analysis. Thirdly, although the quality of included studies was appraised using MMAT and item-level judgments were reported in accordance with methodological guidance, a sensitivity analysis was not applied in the present study representing a limitation. In fact, assessing the robustness of findings in relation to methodological decisions could have strengthened the certainty of evidence. Considering this limitation, future research should include sensitivity analysis to enhance the rigor and transparency of evidence synthesis. For these reasons, as this review represents the first component of a broader research program including also an umbrella review, this additional analysis will be implemented in the subsequent stage to strengthen the quality of evidence. Finally, most included studies relied on self-reported outcomes and cross-sectional designs, which may limit causal inference and generalizability. Despite these limitations, this review provides an up-to-date and extensive synthesis of contemporary evidence in elite athlete career transition and mental health support systems.

## Figures and Tables

**Table 1:** List of the included studies and geographical representation.

Ref.	Year	Authors	Research Context	Country
[49]	2015	Gulliver et al.	Mental Health support to elite athletes	Australia
[50]	2015	Tshube et al.	Career transition out of sport and retirement	Botswana, USA
[51]	2015	Stronach et al.	Career transition out of sport and retirement	Australia
[52]	2015	Gouttebarge et al.	Mental Health support to elite athletes	Netherlands
[53]	2015	Cosh et al.	Career transition out of sport and retirement	Australia
[44]	2015	Torregrosa et al.	Career transition out of sport and retirement	Spain
[54]	2016	Vilanova et al.	Career transition out of sport and retirement	Spain
[55]	2016	Ronkainen et al.	Career transition out of sport and retirement	China, Denmark, Finland
[56]	2016	Gouttebarge et al.	Mental Health support to elite athletes	Netherlands
[57]	2016	Newman et al.	Career transition out of sport and retirement	United Kingdom
[58]	2017	Zurc	Mental Health support to elite athletes	Slovenia
[59]	2017	Hardy et al.	Career transition out of sport and retirement	United Kingdom
[60]	2017	Brown et al.	Career transition out of sport and retirement	South Africa
[61]	2017	Vilanovava et al.	Career support	Spain
[62]	2017	Kuettel et al.	Career transition out of sport and retirement	Switzerland, Denmark, Poland
[63]	2017	Biggin et al.	Mental Health support to elite athletes	United Kingdom
[64]	2017	Gouttebarge et al.	Mental Health support to elite athletes	Netherland
[65]	2018	Howells et al.	Mental Health support to elite athletes	United Kingdom
[66]	2018	Tshube et al.	Career transition out of sport and retirement	Botswana, USA
[67]	2018	Bundon et al.	Career transition out of sport and retirement	Canada
[68]	2018	Brown et al.	Career transition out of sport and retirement	United Kingdom
[69]	2018	Carapinheira et al.	Career transition out of sport and retirement	Portugal
[70]	2018	Demetriou et al.	Mental Health support to elite athletes	Australia
[71]	2018	Moret et al.	Career transition out of sport and retirement	Switzerland
[18]	2018	Barriopedro et al.	Career transition out of sport and retirement	Spain
[72]	2018	Ryan	Athletic identity and retirement	New Zealand
[73]	2018	Papathomas	Career transition out of sport and retirement	United Kingdom
[74]	2018	Hong et al.	Career transition out of sport and retirement	United Kingdom
[75]	2018	Blackett et al.	Career transition out of sport and retirement	United Kingdom
[76]	2019	Brown et al.	Athletic identity and retirement	United Kingdom
[77]	2019	Hallmann et al.	Career transition out of sport and retirement	Germany
[78]	2019	Lebrun et al.	Mental Health support to elite athletes	United Kingdom
[79]	2019	Kruyt et al.	Mental Health support to elite athletes	South Africa
[80]	2019	Kola-Palmer et al.	Mental Health support to elite athletes	United Kingdom
[81]	2020	Raabe et al.	Mental Health support to elite athletes	USA
[82]	2020	Dunne et al.	Mental Health support to elite athletes	USA
[83]	2020	Lundqvist	Career transition out of sport and retirement	Sweden
[84]	2020	Oulevey et al.	Mental Health support to elite athletes	Japan
[85]	2020	Holding et al.	Mental Health support to elite athletes	USA
[86]	2020	McLoughlin et al.	Mental Health support to elite athletes	United Kingdom
[87]	2020	de Subijana et al.	Career support	Spain
[88]	2020	de Subijana et al.	Career transition out of sport and retirement	Spain
[89]	2020	Kola-Palmer et al.	Mental Health support to elite athletes	United Kingdom
[90]	2020	Smismans et al.	Career transition out of sport and retirement	Italy, Spain, Belgium, Germany, Slovenia, Sweden
[91]	2020	de Subijana et al.	Career transition out of sport and retirement	Spain
[92]	2020	Cosh et al.	Career transition out of sport and retirement	Australia
[93]	2020	Blackett et al.	Career transition out of sport and retirement	United Kingdom
[94]	2021	Poucher et al.	Mental Health support to elite athletes	Canada, Australia
[95]	2021	Kuettel et al.	Mental Health support to elite athletes	Denmark
[96]	2021	Ramos et al.	Career transition out of sport and retirement	Portugal
[97]	2021	Ojio et al.	Mental Health support to elite athletes	Japan
[98]	2021	Yongrui et al.	Career transition out of sport and retirement	China
[99]	2021	Buckley et al.	Career transition out of sport and retirement	Australia
[100]	2021	Hong et al.	Career transition out of sport and retirement	United Kingdom
[101]	2021	Moreno et al.	Career transition out of sport and retirement	Spain
[102]	2021	Lundqvist et al.	Mental Health support to elite athletes	Sweden
[103]	2021	Chroni et al.	Career transition out of sport and retirement	Norway
[104]	2021	Van Patten et al.	Mental Health support to elite athletes	USA
[105]	2021	Silver	Mental Health support to elite athletes	Canada
[106]	2021	Oltmans et al.	Mental Health support to elite athletes	Netherlands
[107]	2021	Aitchison et al.	Career support	United Kingdom
[108]	2021	Barnes et al.	Career support	United Kingdom
[109]	2022	Bennett et al.	Mental Health support to elite athletes	Canada, USA
[110]	2022	Jones et al.	Athletic identity and retirement	United Kingdom, Canada
[111]	2022	Åkesdotter et al.	Mental Health support to elite athletes	Sweden, Canada
[112]	2022	Hume et al.	Mental Health support to elite athletes	New Zealand
[113]	2022	Hickey et al.	Career transition out of sport and retirement	Switzerland, United Kingdom
[114]	2022	Brassard et al.	Career transition out of sport and retirement	Canada
[115]	2022	Perry et al.	Mental Health support to elite athletes	United Kingdom
[116]	2022	Robnik et al.	Career transition out of sport and retirement	Slovenia
[117]	2022	Kaski et al.	Mental Health support to elite athletes	Finland
[118]	2022	Hong et al.	Career support	United Kingdom
[119]	2022	Schmid et al.	Career support	Switzerland
[120]	2022	Trudel et al.	Career transition out of sport and retirement	Canada
[121]	2022	McLoughlin et al.	Mental Health support to elite athletes	United Kingdom
[122]	2022	Jovanovic et al.	Mental Health support to elite athletes	Slovenia
[123]	2023	Yufeng et al.	Career transition out of sport and retirement	China
[124]	2023	Couch et al.	Mental Health support to elite athletes	USA
[125]	2023	Sauvé et al.	Career support	Canada
[126]	2023	Willson et al.	Mental Health support to elite athletes	Canada
[127]	2023	Hong et al.	Career transition out of sport and retirement	United Kingdom
[128]	2023	Schmid et al.	Athletic identity and retirement	Switzerland
[129]	2023	Parrott S	Mental Health support to elite athletes	USA
[130]	2023	Hong et al.	Career transition out of sport and retirement	United Kingdom
[131]	2023	Brassard et al.	Career support	Canada
[132]	2023	Miller et al.	Career transition out of sport and retirement	United Kingdom
[133]	2023	Oforeh et al.	Mental Health support to elite athletes	USA
[134]	2023	Oguro et al.	Mental Health support to elite athletes	Japan
[135]	2023	Poucher et al.	Career transition out of sport and retirement	Canada
[42]	2023	Hong et al.	Career support	United Kingdom
[136]	2023	Röthlin et al.	Mental Health support to elite athletes	Switzerland
[137]	2023	Geiger et al.	Mental Health support to elite athletes	Germany
[138]	2024	Brockett et al.	Mental Health support to elite athletes	Australia
[139]	2024	Haslam et al.	Career transition out of sport and retirement	Australia, Belgium, United Kingdom
[140]	2024	Thornton et al.	Career transition out of sport and retirement	USA, Canada
[141]	2024	Edlund et al.	Mental Health support to elite athletes	Sweden
[142]	2024	Teixeira et al.	Career transition out of sport and retirement	Portugal
[4]	2024	Küttel et al.	Mental Health support to elite athletes	Sweden
[143]	2024	Lane	Mental Health support to elite athletes	Australia
[144]	2024	Hlasová et al.	Athletic identity and retirement	Switzerland
[145]	2024	Schmid et al.	Athletic identity and retirement	Switzerland
[146]	2024	Mooney et al.	Mental Health support to elite athletes	United Kingdom
[147]	2024	Young et al.	Career transition out of sport and retirement	Australia
[148]	2024	Murdoch et al.	Career transition out of sport and retirement	United Kingdom
[149]	2024	Bilgoe et al.	Mental Health support to elite athletes	Netherlands
[150]	2024	Dallam et al.	Mental Health support to elite athletes	USA
[151]	2024	Bu et al.	Mental Health support to elite athletes	China
[152]	2024	Prior et al.	Mental Health support to elite athletes	United Kingdom
[153]	2024	Brown	Career transition out of sport and retirement	United Kingdom
[154]	2024	Rohlfs et al.	Mental Health support to elite athletes	Brazil
[155]	2025	Farello et al.	Mental Health support to elite athletes	USA
[156]	2025	Browne et al.	Mental Health support to elite athletes	United Kingdom
[157]	2025	Prior et al.	Mental Health support to elite athletes	United Kingdom
[158]	2025	Steinfeldt et al.	Mental Health support to elite athletes	Germany
[159]	2025	Papathomas et al.	Mental Health support to elite athletes	United Kingdom, USA, Switzerland, Sweden, Canada, Singapore, Fiji, Latvia, and Italy
[160]	2025	Willson et al.	Mental Health support to elite athletes	Canada
[161]	2025	Liu et al.	Mental Health support to elite athletes	China

**Table 2:** Synthetic overview of the research contexts, general study outcomes, recommendations for support strategies and service implementation, online applicability, and sustainability considerations identified in the present study.

Ref.	Research Context	Synthetic Outcomes of the Included Studies	Outcomes Relevant for Digital Resources Implementation	Applicability to Digital Resources	Sustainability Takeaways
[72,76,110,128,144,145]	Athletic identity and retirement	▪ Athletic identity dominates elite athletes’ lives and challenges post-retirement well-being. ▪ Successful adjustment is linked to retirement planning, self-complexity, and supportive relationships. ▪ Family and reflective practices foster smoother adaptation.	▪ Emphasize balanced personal development and identity diversification. ▪ Provide resources for families, partners, and long-term reflective support. ▪ Encourage tailored transition planning and post-retirement engagement.	▪ Interactive modules for identity work and career planning. ▪ Family involvement, journaling and mentoring spaces. ▪ Workshops, and self-reflection tools.	▪ Holistic and proactive support enhances athletes and their family well-being. ▪ Promotes ongoing engagement and digital platform.
[42,61,87,107,108,118,119,125,131]	Career support	▪ Most effective when integrating psychological, educational and vocational elements. ▪ Dual-career planning and mentoring enhance resilience and employability. ▪ Main challenges: stigma, unequal access, limited organizational involvement.	▪ Integrate dual career, financial literacy, and psychological support. ▪ Facilitate personalized guidance and self-assessment tools. ▪ Include practitioners and coaches in best-practice sharing.	▪ Provide adaptive pathways combining mental well-being, career, and transition resources. ▪ Engage families and coaches; promote continuous evaluation.	▪ Personalization, adaptive learning, and multi stakeholder engagement strengthen long-term sustainability.
[18,44,50,51,53,54,57,59,60,62,66,67,68,69,71,73,74,75,77,83,88,90,91,92,93,96,98,99,100,101,103,109,110,113,114,116,120,123,127,130,132,135,139,140,142,147,148,153]	Career transition out of sport and retirement	▪ Retirement involves psychological, social, financial, and cultural factors. ▪ Early preparation and dual career support are key proactive factors. ▪ Barriers: stigma, performance culture, limited access, and financial insecurity.	▪ Offer integrated and Customizable digital services (career, mental health, identity). ▪ Include early intervention and literacy modules. ▪ Facilitate mentoring, family/peer/community engagement, and inclusion of vulnerable groups.	▪ Modular online pathways for career and mental health support. ▪ Interlinked practitioner, family, and community networks. ▪ Tailored digital guidance across contexts.	▪ Continuous, inclusive, and collaborative systems sustain athlete well-being. ▪ Ongoing evaluation and stakeholder synergy reinforce platform durability.
[4,49,54,56,58,63,64,65,70,78,79,80,81,82,84,85,86,89,94,95,97,102,104,105,106,109,111,112,115,117,121,122,124,126,129,133,134,136,137,138,141,143,146,149,150,151,152,154,155,156,157,158,159,160,161]	Mental Health support to elite athletes	▪ High prevalence of anxiety, depression, eating disorders, and mental distress. ▪ Risk factors: injury, identity foreclosure, stigma, lack of early intervention. ▪ Protective factors: literacy, social support, autonomy-supportive settings. ▪ Persistent gaps for women, minorities, and retired athletes.	▪ Integrate screening, literacy, and stigma reduction tools. ▪ Ensure confidential access to professional support. ▪ Include trauma-informed and culturally sensitive interventions.	▪ Develop modular support journeys with self-assessment and professional follow-up. ▪ Build inclusive, psychologically safe environments. ▪ Enable collaboration with practitioners, coaches, and families.	▪ Sustainability requires continuous monitoring, stakeholders’ collaboration, and adaptability. ▪ Addressing gender, culture, and sport-specific risks ensures long-term relevance.

## Data Availability

No new data were created or analyzed in this study. Data sharing is not applicable to this article.

## Supplementary Materials

The following supporting information can be downloaded at: https://www.mdpi.com/2075-4663/13/12/438/s1. Supplementary Materials File S1: PRISMA 2020 checklist; Supplementary Materials File S2: Search String for each database; Supplementary Materials File S3: Characteristics of included studies; Supplementary Materials File S4: Mixed Methods Appraisal Tool (MMAT) checklist items.
