# Supplementary material for: Sustainable Career Transitions and Mental Health Support in Elite Sport: A Systematic Review of Evidence and Practices
Source: Sports (Basel). 2025 Dec 5;13(12):438. doi: 10.3390/sports13120438 (PMC12813639; doi:10.3390/sports13120438)
Supplement: Supplementary file 1 [file sports-13-00438-s001.zip › 2_Supplementary File S2_Search strategies and list of excluded studies presented in alphabetic order.pdf]

Supplementary File S2. Search strategies and list of excluded studies presented in alphabetic order.

Database	Search String (exact syntax)	Filter / Fields	Last search/ Alert Period
**SPORTDiscus (EBSCOhost)**	((“high-performance athletes” OR “elite athlete” OR “Olympic athlete*”) AND (“career transition” OR “career transitions”) AND (“mental health” OR “psychological health” OR “support services”))	Document type: Article; Language: English. Publication years: 2015 – 2025	Primary search: 10 March 2025. Automatic alert active until 31 May 2025 (no additional results)
**PsycINFO**	((“high-performance athletes” OR “elite athlete” OR “Olympic athlete*”) AND (“career transition” OR “career transitions”) AND (“mental health” OR “psychological health” OR “support services”))	Document type: Article; Language: English. Publication years: 2015 – 2025	Primary search: 10 March 2025. Automatic alert active until 31 May 2025 (no additional results)
**Scopus**	TITLE -ABS-KEY ((“high-performance athletes*” OR “elite athlete*” OR “Olympic athlete*”) AND (“career transition” OR “career transitions”) AND (“mental health” OR “psychological health” OR “support services”))	Document type: Article; Language: English. Publication years: 2015 – 2025	Primary search: 10 March 2025. Second search: at the end of May 2025 (no additional results)
**Web of Science**	TS= ((“high-performance athletes*” OR “elite athlete*” OR “Olympic athlete*”) AND (“career transition” OR “career transitions”) AND (“mental health” OR “psychological health” OR “support services”))	Document type: Article. Language: English. Publication years: 2015 – 2025	Primary search: 10 March 2025. Second search: at the end of May 2025 (no additional results)
**Google Scholar**	(“high-performance athletes career transitions” OR “elite athletes career transitions” OR “Olympic athletes career transitions”) AND (“high-performance athletes’ mental health” OR “elite athletes’ mental health” OR “Olympic athletes’ mental health”) AND (“high-performance athletes’ psychological health” OR “elite athletes’ psychological health” OR “Olympic athletes’ psychological health”) AND (“high-performance athletes’ support services” OR “elite athletes support services” OR “Olympic athletes support services”)	Sorted by relevance. English language. Publication years: January 2015 – May 2025	Primary search: 10 March 2025. Automatic alert active until 31 May 2025 (no additional results)

Authors	Exclusion Criteria
Åkesdotter et al.	Out of scope, competition level uncertain
Barker-Ruchti et al.	Out of scope
Beable et al.	Abstract, not original article
Bennie et al.	Out of scope
Bergström et al.	Out of scope
Beth Aitchison	Out of scope
Biggins et al.	Out of scope
Bindra	Q1 and Q2 of MMAT were "No"
Bu et al.	Mixed competition level and data for the sample of interest was not presented
Burns et al.	Out of scope
Chambers et al.	Out of scope, competition level uncertain
Chroni, et al.	Out of scope
Colagrai et al.	Not English language
Cosh et al.	Out of scope
Cuvelier et al.	Out of scope
Davies et al.	Out of scope
Donnelly et al.	Out of scope
Edwards	Out of scope
Giffin et al.	Out of scope
Gorczynski et al.	Methodology insufficient
Haslam et al.	Out of scope, competition level uncertain
Henriksen et al.	Data for the sample of interest was not presented
Hilpisch et al.	Out of scope
Juhász et al.	Out of scope
Kenny	Methodology insufficient
Kola-Palmer et al.	Out of scope
Kuettel et al	Out of scope
Kurniati	Out of scope
Lebrun et al.	Out of scope
Lundqvist et al.	Out of scope
Marin-Urquiza et al.	Out of scope
Martinez-Pascual et al.	Out of scope
Montero et al.	Out of scope
Mthombeni et al.	Out of scope
Nicholls et al.	Out of scope
Oevreboe et al.	Out of scope
Perry et al.	Out of scope
Pino et al.	Not elite/high performance sample
Plateau	Out of scope
Purcell et al.	Out of scope
Rice et al.	Q1 and Q2 of MMAT were "No"
Rice et al.	Out of scope, validation of a tool
Ronkainen et al.	Out of scope
Röthlin et al.	Methodology insufficient
Ryba et al.	Out of scope
Schinke et al.	Book chapter, not original article
Sebbens et al.	Out of scope
Tachiya	Brief report, not original article
Tekavc et al.	Out of scope
Thornton et al.	Out of scope
Timpka et al.	Out of scope
Wang et al.	Out of scope, competition level uncertain
Woodford et al.	Out of scope
Wylleman et al.	Book chapter, not original article
Zaré et al.	Out of scope
