# Supplementary material for: Sustainable Career Transitions and Mental Health Support in Elite Sport: A Systematic Review of Evidence and Practices
Source: Sports (Basel). 2025 Dec 5;13(12):438. doi: 10.3390/sports13120438 (PMC12813639; doi:10.3390/sports13120438)
Supplement: Supplementary file 1 [file sports-13-00438-s001.zip › 3_Supplementary File S3_ characteristics of the included studies.pdf]

Supplementary file S3. Characteristics of the included studies (e.g., year of publication, purpose, participants' characteristics, methodologies and tools, variables), presented in alphabetic order.

Note: ND = not declared; Qual = qualitative; Quant = quantitative; Mix = Mixed-Methods; Quest = questionnaire.

Year	Author	Purpose	Sample	Sample size	Gender	Type of Sport	Methodology	Variables	Instruments/Tools	Confidence of evidence
2021	Aitchison et al.	To explore the experiences of social support in elite British para-swimmers and the influence on their wellbeing and performance.	Para-elite	n=8	Mix	Individual	Qual	Perceptions of social support; Support network members; Effects on well-being and performance	Semi-structured interviews	Moderate
2022	Åkesdotter et al.	To evaluate the prevalence of psychiatric disorders among treatment-seeking elite athletes (EA) or high-performance coaches (HPC) in psychiatric outpatient settings	Elite Athletes and high-performance coaches	Elite Athletes n=221; High-Performance Coaches n=34	Mix	Multisport	Quant	Anxiety/Affective/Eating/Mental and Behavioural disorders, Substance use, Comorbidity	Mini-International Neuropsychiatric Interview (MINI) 7.0.0 Swedish version	High
2021	Barnes et al.	To explore growth following the experience of stressors and compare the experiences of elite athletes who exhibit higher and lower levels of growth.	Elite athletes	n=6 (n=3 male and n=3 female)	Mix	Multisport	Qual	Events, experiences, and states in relation to growth, stressors and growth-related experiences	Semi-structured interviews	Moderate
2018	Barriopedro et al.	To evaluate if perceived difficulties during the retirement transition varied according to the voluntariness of the decision, planning of a post sport career life, satisfaction with the sport achievements, and career path, and to analyze if these perceived difficulties varied according to the type of the student-athlete's DC trajectory.	Former elite athletes	n=477 (n=298 male; n=179 female)	Mix	Multisport	Quant	Sport profile, sociodemographic profile, academic profile, employment, the retirement process, and current relationship with sport, the type of career path, involuntariness/voluntariness as well as planning retirement in advance, the degree of satisfaction related to the athletic achievements, difficulties perceived by athletes during the transition out of sport in seven different areas (professional career out of sport, studies, family, social networking, leisure, health, and economy)	Quest.	Moderate

Year	Author	Purpose	Sample	Sample size	Gender	Type of Sport	Methodology	Variables	Instruments/Tools	Confidence of evidence
2022	Bennett et al.	to explore how Canadian elite athletes perceived, experienced, and coped with the Covid-19 pandemic and postponement of the Tokyo 2020 Games.	Olympic/Paralympic athletes	n=21 (7 Paralympic athletes; 14 Olympic athletes)	Mix	Multisport	Qual	Characteristics/Behaviours; Sport Psychology; Stress; Psychological growth	Semi-structured interviews analyzed using reflexive thematic analysis	Moderate
2017	Biggin et al.	To further investigate the role of coaches and athletes in the context of mental ill-health and explore some of the challenges to promoting the recommendations made by MIND.	Coaches and elite athletes	n=35 (n=19 elite athletes and n=16 coaches)	Mix	Multisport	Qual	Mental health problems	three-round Delphi method: sequential questionnaires	High
2024	Bilgoe, et al.	To assess prevalence and contributing factors for mental health symptoms among Dutch elite athletes and their coaches	Elite athletes	n=156 athletes, n=95 coaches	Mix	Multisport	Quant	Mental health symptoms, adverse life events, injuries	Quest. (APSQ, GAD-7, PHQ-9, AUDIT-C, ASSQ, BEDA-Q)	Moderate
2018	Blackett et al.	To understand the experiences of elite athletes transitioning into high-performance coaching	Former elite athletes (Football and Rugby)	n=13	Male	Multisport	Qual	Career transition, coach development, identity	Semi-structured interviews	Moderate
2020	Blackett et al.	To analyze how elite male association football and rugby union athletes based in England and Wales (re)created, renegotiated or transformed their identities when negotiating a fast-tracked career pathway into a post-athletic high-performance coaching role.	Elite	n=15	Male	Multisport	Qual	career transition; career trajectory, and in (re)creating a coaching identity	Semi-structured interviews	Moderate
2022	Brassard et al.	To characterize the capability of elite athletes to plan and prepare a career transition out of sport within the context of their environment. In other words, the article explores the real opportunity they have to plan a career they value and to prepare themselves for the transition into that career.	Active elite athletes	n=14	Mix	Multisport	Qual	A) one enabling capabilities to plan and prepare a career transition out of sport, (B) one restricting capabilities to plan and prepare a career transition out of sport, and (C) one hindering capabilities to plan and prepare a career transition out of sport.	Semi-structured interviews	Moderate
2023	Brassard et al.	To predict and define factors associated with help seeking behaviours toward career support resources and identify the	elite athletes (Olympic and Paralympic	n=190; n=57 male; n= 133 female	Mix	Multisport	Quant	Frequency of use of eight different services offered: appointments with an advisor to help the athlete get	Quest.	Low

Year	Author	Purpose	Sample	Sample size	Gender	Type of Sport	Methodology	Variables	Instruments/Tools	Confidence of evidence
		perceived barriers to elite Canadian athletes seeking help.	national team members)					counseling and navigate the resources, online tutoring offered through free access to a platform, career support resources including resume building, mock interviews and job search skills, access to a mentoring program and mental health consultations, access to online webinars on various topics (finance, sponsorship, health, etc.) as well as various workshops (regional workshops, social media workshop, mental health workshop, etc.) and opportunities to sign up for networking activities to meet potential employers. Participants were also asked to specify if they had attended a transition program (a two-day workshop on sport transition) and if they had received a scholarship for the Coaching Education Program certification or for a business program from Queen's University. Perceived barriers and engagement were also measured.		
2024	Brockett et al.	To illuminate the gaps and opportunities in existing support systems and better understand which initiatives may have the greatest benefit in supporting athletes to transition out of elite sport	National/International retired high-performance athletes	n=53 (of 102) completed all measures; n=11 were interviewed	Mix	Multisport	Mixed	retirement support, retirement difficulties, retirement experiences, psychological well-being, psychological distress	Survey and interview	Moderate
2024	Brown	To understand how sociocultural discourses shape how elite coaches construct athletes'	Coaches' Elite athletes	n=8 male elite coaches (all coached	Male	Multisport	Qual	Constructions of athlete retirement, role of coach in transition, power relations,	Semi-structured interview guide;	Moderate

Year	Author	Purpose	Sample	Sample size	Gender	Type of Sport	Methodology	Variables	Instruments/Tools	Confidence of evidence
		retirement transitions and their own roles within this process.		Olympic/world level athletes)				identity negotiation, support practices, organizational constraints	thematic and discourse analysis (Willig, 2013)	
2017	Brown et al.	To compare mental health between former professional rugby players who were and weren't forced to retire.	Professional Rugby players from three countries	n=293	Male	Team Sport	Qual	Distress; anxiety/depression; Sleeping disturbance; alcohol misuse	Quest.	Low to moderate
2018	Brown et al.	To gain a more comprehensive insight into the ways that social support may in hence how athletes adjust to life following retirement from elite sport.	Former elite athletes	n=1 major Championship; n=7 competed at the Olympic Games	Mix	Multisport	Qual	social support, identity, and transition outcomes.	Semi-structured interview	Low to moderate
2019	Brown et al.	To explore the experiences of parents and partners of elite athletes during the athletes' transition out of sport. Specifically, an interpretive phenomenological approach was Used to explore the interpersonal nature of transition and the way (s) that retirement from sport can affect close relationships. The present research also aimed to understand how parents and partners of athletes managed and interpreted their role in the process of transition, including their possible role as providers of support.	Former elite athletes	n=7	Mix	Multisport	Qual	This interpretation of the parents and partners' accounts is captured by an overarching theme that was labelled parallel and shared experiences of transition'. This overarching theme provided the context for three other interwoven themes that form the structure of the analysis that follows: (a) initial experiences of transition, (b) experiences of being a supporter, and (c) integrating experiences into current life.	Semi-structured interviews	Moderate
2025	Browne et al.	To assess the relationship between organizational stressors and mental health and wellbeing indicators of the Irish Olympic Team, pre- and post- the 'Tokyo 2020' Olympic Games; (b) to also examine the 5 differences in mental health and wellbeing between the athletes and staff of Team Ireland pre and post Games	Olympic Team (athletes and team staff)	T1: n=98; T2: n=70 (all staff) T1: n=32; T2: n=33 (athletes)	Mix	Multisport	Mixed	Goals and Development; Logistics and Operations; Team and Culture; Coaching; Selection	Survey and Organizational Stressor Indicator for Sport Performers (OSI-SP), the Mental Health Continuum-Short Form (MHC-SF), and the Sport Mental Health Assessment Tool-1 (SMHAT-1).	High
2024	Bu et al.	To understand the mental health experiences, MHL and health-seeking behaviours of Chinese	Elite	n=37 (20 athletes)	Mix	Multisport	Qual	Experiences of mental health, Mental health literacy, Help-seeking attitudes, Help-	Interviews	High

Year	Author	Purpose	Sample	Sample size	Gender	Type of Sport	Methodology	Variables	Instruments/Tools	Confidence of evidence
		elite athletes from the triangulated perspectives of elite athletes, coaches, and sport team officials.						seeking intentions, Help-seeking behaviours, Chinese sports environment, Chinese cultural background		
2021	Buckley et al.	To explore how COVID-19 affected disordered eating and body image among current and former athletes, with implications for transitional phases such as retirement	Elite athletes	n=204 (93 current; 111 former)	Mix	Multisport	Mixed	Body image, food relationship, exercise, identity	Quest. (EAT-26, self-report COVID-19 impact items9	High
2018	Bundon et al.	To explore how the current organization and delivery of Paralympic sport are informing the retirement experiences of elite para-athletes by answering the following research questions: (1) How are elite para-athletes transitioning out of sport and into employment or education? And (2) How specifically do disability and/or impairment impact upon this transition?	Paralympians and elite para-athletes	n=60; n=48 Paralympians (n=17 one Games, n=17 two Games, n=7 three Games, n=7 four or more Games); n=21 won medals at Paralympic games; n=12 represented Great Britain internationally	Mix	Multisport	Qual	(1) the multiple, concurrent and complex reasons elite para-athletes leave sport, (2) the increasing professionalization of para-sport and the implications on when, why and how para-athletes retire, (3) the challenges and discrimination para-athletes face in leaving para-sport and entering the workforce, and (4) suggestions to better prepare para-athletes for their transition out of elite sport.	Survey and semi-structured interviews	High
2018	Carapinheira et al.	To explore the process of career termination of elite soccer players, comparing the quality and the resources to support career termination over the last three decades.	Former elite Portuguese soccer players	n=90 (n=30 retired between 1985-1995 Group I, n=30 retired between 1996-2005 Group II, n=30 retired between 2006-2015 Group III)	Male	Team Sport	Mixed	The questionnaire was defined by four sections: (a) biographical data, (b) athletic career, (c) quality of career termination	Quest.	Low to moderate
2021	Chroni et al.	To explore what sport federations look for when recruiting elite athletes into coaching, and what resources are offered to retiring elite athletes transitioning to coaching careers.	Elite athletes in Norway transitioning to coaching (indirect sample: 10 federation	n=10 federation officials representing 8 sports (recruiting retiring elite athletes)	NA	Multisport	Qual	Recruitment criteria, support resources, transition challenges, phases of transition (career shift, reidentification, professional development), support timing	Semi-structured interview guide, Braun & Clarke's thematic analysis	Moderate

Year	Author	Purpose	Sample	Sample size	Gender	Type of Sport	Methodology	Variables	Instruments/Tools	Confidence of evidence
			officials, 8 sports)							
2015	Cosh et al.	To examine the career transitions and post-career wellbeing of two athletes (Ian Thorpe and Grant Hackett) within a socio-cultural context, as evidenced by the mass media. Through examining the post-career experience, a better understanding of post-career wellbeing and the contributing factors can be garnered.	Retired athletes	121 media articles on n=2 former Olympic athletes during retirement	Male	Individual	Qual	Patterns of how identity and causality for transition difficulties were depicted and the implications that these versions serve to perpetuate for retiring athletes	Discursive analysis of selected media extracts	Low to moderate
2020	Cosh et al.	To explore crisis transitions and related psychological distress within a cultural context	Former elite Australian athletes	n=9	Mix	Multisport	Qual	Crisis transition; retirement experience; Mental health outcomes; Cultural discourses and representations; Athletic identity and self-worth; Support systems / Career assistance	Single host interviewing	Moderate
2023	Couch et al.	To bring to voice and position US African American female Olympians lived experiences of selected identity components (i.e., race, gender, athletic identity) as they navigated specific transitions during their competitive careers.	US African American Olympic athletes	n=10	Female	Individual	Qual	Identity phases: (i) Initial track and field experience: early identity-I'm Black, female, and fast; (ii) University experience: Black student-athlete-the only one; (iii) Transition to Olympic and professional competition: Black female identity-I'm an Olympian; (iv) Professional and post-Olympic experience	Semi-structured interviews	Moderate
2024	Dallam et al.	To assess the prevalence of IV in elite sport and its association with mental health outcomes	Elite U.S. Olympic athletes	n=473	Mix	Multisport	Quant	IV types, mental health diagnoses, eating disorders	Adapted NatSCEV, Campus Climate Survey	Moderate
2020	de Subijana et al.	To compare the athletic career and retirement of former elite athletes according to the sport practiced and to provide information for the athletes' counselors. Furthermore, describing the retirement transition of former elite athletes may help practitioners to aid the next generation of athletes at some key points to deal successfully	Former elite athletes	n=410	Mix	Multisport	Quant	23 variables were considered: gender, the type of sport, the events of the career path (age of starting practicing the sport, age of entering the elite level, and age of maximum sport performance), the hours the athlete trained per week at the elite level), their career path during their mastery stage; the	Quest.	Moderate

Year	Author	Purpose	Sample	Sample size	Gender	Type of Sport	Methodology	Variables	Instruments/Tools	Confidence of evidence
		with their transitions. Therefore, it could be interesting to analyze in-depth the athletic careers of former elite athletes and shed some light on their retirement and current lifestyles.						sport retirement features, if the sport retirement process was planned, gradual and voluntary; if their working and economic situation was solved at retirement; the athletes' level of studies at their retirement; their working status; their relation with sport nowadays (I do physical activity or practice sport; I compete in veterans events; I keep in touch with my coaches; I have an employment related to sport; I attend sport events as a spectator; I informally counsel young athletes); if they practiced sport or physical activity, they answered about the frequency and how many hours they spent each session.		
2020	de Subijana et al.	To explore the learning context and its relationship with life skills in former elite athletes	International former elite athletes	n=477; n=298 male; n=179 female	Mix	Multisport	Quant	Athlete's current age (years); the athlete's level of studies at sport retirement as well as the current level of studies; the type of sport (1 = individual; 2 = opposition; 3 = team); the age the athlete started practicing this sport; the age the athlete began competing at the elite level; the number of hours the athlete trained per week at the mastery stage; the athlete's age at retirement; whether the retirement process was gradual/radical, voluntary/involuntary, and planned/not planned; whether the athlete was working or not	Quest.	Moderate to high

Year	Author	Purpose	Sample	Sample size	Gender	Type of Sport	Methodology	Variables	Instruments/Tools	Confidence of evidence
2020	de Subijana et al.	To describe the work integration after retirement in elite athletes; to compare their working integration of women and men, and in Olympic and non-Olympic athletes and to specify the factors that affect their employment status, and their current monthly income.	Former elite athletes	n=477 (n=298 male; n=179 female)	Mix	Multisport	Quant	Sociodemographic profile, sport profile, academic profile, employment, the retirement process and their current lifestyle	Quest.	Moderate
2018	Demetriou et al.	To explore a negative case study of an athlete recently retired from a team sport (Australian Rules Football) in order to generate knowledge on how to improve the retirement process.	Elite athlete	n=1	Male	Team Sport	Qual	Identity, well-being, grief, coping, communication	Semi-structured interviews	Low to moderate
2020	Dunne et al.	To describe how reasons for prescription opioid use while in the National Football League corresponds to use and misuse in retirement	Former National Football League Players	n=336	Male	Team Sport	Quant	prescription opioids, drug use, health	2009 Retired NFL Players Association Directory (secondary analysis)	Moderate
2024	Edlund et al.	To explore changes in symptoms of eating disorders, compulsive exercise, and depression, between two assessments 12 months apart, among elite gymnasts.	Swedish national team of gymnastics	n=94	Mix	Individual	Quant	Changes in exercise for weight control, body dissatisfaction, avoidance and rule-driven behavior and depression	Eating Disorders Inventory 3 (EDI 3), Compulsive Exercise Test, Montgomery-Asberg Depression Rating Scale-Self report (MADRS-S)	Moderate to high
2025	Farello et al.	To explore the nuanced trajectory of mental health across the sporting careers of professional female athletes, utilizing a three-part narrative history	Professional	n=9 female professional athletes	Female	Multisport	Qual	Athletic identity phases: development, foreclosing, conflict and rising action, climax/rupture, resolution/reconstruction	Interviews: the first two were unstructured, the third semi-structured	Moderate
2023	Geiger et al.	To assess data on mental health of elite athletes and investigate associations and interconnections among different variables using network analysis.	Elite	n=275	Mix	Multisport	Quant	sociodemographic data, including age, sex, family status, housing situation, level of education, financial situation, and occupation	Survey (digital)	Moderate
2015	Gouttebarge et al.	To determine the prevalence of mental health problems and psychosocial difficulties in current and former professional	Current and former professional	n=253	Male	Team Sport	Quant	GHQ-12, Distress Screener, Maslach Burnout (UBOS), Rosenberg Self-Esteem, AUDIT-C, and SARRS.	Quest.	Moderate

Year	Author	Purpose	Sample	Sample size	Gender	Type of Sport	Methodology	Variables	Instruments/Tools	Confidence of evidence
		footballers, and to explore the association between psychosocial stressors and the health conditions studied.	football players							
2016	Gouttebarge et al.	To determine the prevalence and comorbidity of symptoms of common mental disorders (distress, anxiety/depression, sleep disturbance, eating disorders, adverse alcohol use) among current and former Dutch elite athletes, and to explore the inference between potential risk indicators (severe injury, surgery, life events, sport career dissatisfaction, social support) and the outcomes measures under investigation.	Current and former elite athletes from the Netherlands Olympic Committee*Netherlands Sports Confederation	n=485 (n=203 current, n=282 former)	Mix	Multisport	Quant	risk indicators and presence of comorbidity symptoms (distress, anxiety)	Quest.	Moderate
2017	Gouttebarge et al.	To (i) establish the 12-month incidence of symptoms of common mental disorders (CMD; distress, anxiety/depression, sleep disturbance, adverse alcohol use, eating disorders) among Dutch elite athletes and (ii) explore their potential association with several stressors (being injured, recent life events, career dissatisfaction)	Elite	n=726	Mix	Multisport	Quant	Injuries; Recent life events; Career dissatisfaction	Quest.	Moderate
2014	Gulliver et al.	To investigate Australian elite athletes' symptoms of general psychological distress and common mental disorders.	Elite	n=224	Mix	Multisport	Qual	Relationship status; Current injury status; Recent injury (last 4 weeks); Relocation for sport; Time since relocation; Education level; Current study status; AIS athlete status; Full-time salary from sport;	Survey	Moderate
2019	Hallmann et al.	To provide insights into the functions and benefits of mentoring in the transition of elite athletes into a professional career	Elite athletes	n=197	Mix	Multisport	Quant	Five mentoring functions present in mentoring relationships in sport ('Benefactor', 'Counsellor', 'Supportive Friendship', 'Role Model' and 'Acceptance'); the impact of the mentoring	Questionnaire	Low to moderate

Year	Author	Purpose	Sample	Sample size	Gender	Type of Sport	Methodology	Variables	Instruments/Tools	Confidence of evidence
								functions on the perceived satisfaction with the support for the transition process		
2017	Hardy et al.	To explore psychosocial aspects of development and careers of athletes, their coaches, and one of their parents	Elite Great Britain athletes	n=32	Mix	Multisport	Qual	Achievement; Career turning point; Behaviours: mastery/outcome focus, total preparation for competition, counterphobia, importance of sport; Ruthlessness/selfishness, Obsessiveness/perfectionism, Need for success; Experiences: Early negative life event plus positive sport event	In-depth interviews	High
2024	Haslam et al.	To explore athletes' negotiation of identity change with a focus on retirement.	Elite athletes	n=21	Mix	Multisport	Qual	Identity, sport retirement	Semi-structured interviews	Moderate to high
2022	Hickey et al	To explore the role of Education and Welfare Officers (EWOs) in supporting English Premier League footballers during their career transitions and retirement	Elite professional football players (English Premier League)	n=15 (n=10 released players and n=5 EWOs)	Mix	Team Sport	Qual	Career transition experiences, identity loss, mental health, coping strategies, social support, possible selves, organizational support	Semi-structured interviews, vignette technique	Moderate to high
2024	Hlasová et al.	To uncover the processes of narrative change in retirement from elite sport. the aim is to understand how elite athletes experience the retirement process, reconstruct their identities, and potentially connect in a meaningful way to new careers.	Elite athlete	n=1	Male	Individual	Qual	central I-positions to demonstrate Archie's narrative-self that is both social and personal. Then, we continue chronologically and, in the section, "Sport Became My Life" highlight some relevant changes and the movement of positions in his early sporting career to introduce what became the dominant narrative for Archie. In the section "Innovation: 'Maybe it would be better if I quit'" we elaborate on the IMs which were coded for to track changes in his dominant narrative and demonstrate	Semi-structured interviews	Low to moderate

Year	Author	Purpose	Sample	Sample size	Gender	Type of Sport	Methodology	Variables	Instruments/Tools	Confidence of evidence
								how Archie went from resisting retirement to transitioning out of sport. Finally, the last section highlights change in the salient voices in Archie's story one year later and demonstrates the on-going process of innovation as he reflects on his career, his retirement and the year that followed.		
2020	Holding et al.	To propose that sport motivation at the career peak and motivation for retirement are important determinants of athletes' disengagement progress from a terminated athletic career and to examine how motivation for retirement and disengagement progress predict retired athletes' well-being.	High-performance athletes	n= 158 (86.1% Olympians; 9.5% Paralympians; 4.4% non-Olympians sport)	Female	Multisport	Quant	motivation for Sport and retirement, disengagement Progress, well-being in retirement	Quest.	Moderate
2018	Hong et al.	To provide an overview of sport career transition support programmes and training and development programmes in 19 countries worldwide as a form of organizational support for both high-performance athletes and practitioners who provide support to high-performance athletes, (2) to present a novel psycho-educational curriculum for practitioners aiming at making an impact on organizational support and management in order to enhance the quality of support services delivered by sporting organizations, and (3) to establish the groundwork for future studies on examining the psycho-educational curriculum by	Sport career transition practitioners and sport psychology experts	n=4 (2 male and 2 female)	Mix	Multisport	Qual	Dual Career Transition Management; Skill Transfer; Social Support; Career Planning	Interview and Quest.	Low

Year	Author	Purpose	Sample	Sample size	Gender	Type of Sport	Methodology	Variables	Instruments/Tools	Confidence of evidence
		indicating significant potential with an exploratory investigation.								
2021	Hong et al.	To investigate high performance athletes' experiences of financial self-management during their athletic career and the organizational support (e.g., career assistance programmes/services) available to them for developing their financial self-management skills and literacy.	Former elite athletes	n=20 (n=10 male; n=10 female)	Mix	Multisport	Qual	(1) sports background; (2) experience as high-performance athletes; (3) career management and planning; (4) challenges and barriers to managing finance and financial wellbeing; (5) development of financial literacy and self-management skills; (6) coping skills/strategies and available external support; and (7) adjustments to post-athletic careers and life generally.	Semi-structured interviews	Moderate
2022	Hong et al.	To identify available organizational support for enhancing athletes' financial literacy and self-management skills in order to share good practice and explore how better to support high performance athletes.	High performance athletes	n=23 (n=21 national in 19 countries and International Olympic Committee (IOC) and Oceania National Olympic Committee (ONOC))	ND	Multisport	Qual	support in financial literacy post sport career	documentary analysis	Moderate
2023	Hong et al.	To offer a comprehensive analysis of Career Assistance Programmes (CAPs) available for high-performance athletes across the globe, addressing the current state of resources for their career development and transitional periods.	Sport organizations	n=23 (5 different countries)	Mix	Multisport	Qual	Responsible organization; Criteria for access; Overall program objectives; Content and activities offered; Who delivers the program; Training of practitioners; Methods of program evaluation (TAB 1)	Web-based data collection (official websites), research tour (field notes and consultations with key officers), deductive content analysis based on the Holistic Athlete Career (HAC) model	Moderate
2023	Hong et al.	To investigate high-performance athletes' experiences of transitioning out of sport and the support provided to them and describes how the findings	Retired high-performance athletes (Olympians, Paralympians	n=20	Mix	Multisport	Qual	causes of retirement, experiences following retirement, career transition distress, and available	Semi-structured interviews	Moderate

Year	Author	Purpose	Sample	Sample size	Gender	Type of Sport	Methodology	Variables	Instruments/Tools	Confidence of evidence
		suggest Corporate Social Responsibility (CSR) initiatives which may inform the development of sport policies addressing issues surrounding sport career transitions.	and Commonwealt h Games)					resources to cope with career transitions.		
2023	Hong et al.	To offer empirical evidence regarding the challenges they face in realizing their Olympic dreams, and to shed light on the transitional challenges, available resources, and needs they face as they move toward post-athletic lives.	Retired Olympians Judokas	n=8	Mix	Individual	Qual	From dreams to Olympic reality; Facing the void: loss of goals and identity; The crucial role of social support; Dual aspects of pre-retirement planning; The double edge of organizational support	Semi-structured interviews	Low to moderate
2018	Howells et al.	To explore the concept of the 'post-Olympic blues' through examining the antecedents of the negative effect experienced following Olympic participation and to articulate whether the post-Olympic blues is a 'normal' short-term phenomenon or whether it is more serious and enduring.	Olympic athletes	n=4	Female	Multisport	Qual	Depression; Depressive symptoms; Olympic experience phases	Interviews investigated through Interpretative Phenomenological Analysis	Moderate
2022	Hume et al.	To investigate differences in self-reported sport injury history and current self-reported health characteristics between former New Zealand rugby and non-contact sport players with a view to identifying issues to be further investigated with stronger epidemiological research designs	National/International former rugby and non-contact sport players	n=470	Male	Team Sport	Quant	Playing Career Injuries and Illnesses, Current Health, Brain health, Alcohol and Other Substance Use	Online Quest. (self-report rugby-sport general health 58-item e-questionnaire)	Moderate
2022	Jones et al.	To explore how retired high-performance athletes renegotiate their relationship to exercise and movement post-retirement, using Foucauldian theory.	Retired elite athletes (authors themselves; former professional soccer players and NCAA runners)	n=3	Mix	Multisport	Qual	Athletic identity, retirement adjustment, exercise re-orientation, discipline and docility	Autoethnographic vignettes, Foucauldian theoretical framework	Moderate

Year	Author	Purpose	Sample	Sample size	Gender	Type of Sport	Methodology	Variables	Instruments/Tools	Confidence of evidence
2022	Jovanovic et al.	To assess mental health issues among elite Slovenian athletes compared to the general population	Elite (Olympic, World, International level)	n=97	Mix	Multisport	Quant	Depression, anxiety, eating disorders, substance use, suicidal ideation	Quest. (PAI, EDI-3)	Moderate
2022	Kaski et al.	To profile elite athlete subjective and psychological mental well-being in conjunction with their self-reported absence and/or presence of mental ill-being, and to explore the relationships between the identified mental well- and ill-being profiles and selected sport-related demands and resources	Elite athletes (active and former)	n=259 (n=93 male; n=166 female)	Mix	Multisport	Quant	mental well-being with the presence of both subjective (life satisfaction) and psychological (positive functioning) well-being; mental ill-being with measures identifying the presence of selected mental health symptoms and maladaptive behaviors (depression, anxiety, possible presence of eating disorders, and risk for alcohol abuse) associated with mental ill-being; sport-related demands and resources	Quest.	Moderate
2019	Kola-Palmer et al.	To examine the direction and strength of a number of correlates related to mental health; namely, psychological stress, athletic identity and attitudes to player welfare managers and policies. The results from the study have the potential of identifying correlates of mental health, which in turn helps develop a comprehensive understanding of rugby league players' mental health and wellbeing, which underpin the development of care and support management, which can in turn facilitate performance gains	Elite Rugby league Players	n=77 (survey 1), n=169 (survey 2)	Male	Team Sport	Quant	Psychological stress, mental health symptoms, athletic identity, attitudes to player welfare	Quest. (MHI-5, PSS-10, AIMS, Player Welfare Quest.)	Moderate
2020	Kola-Palmer et al.	To elucidate factors associated with actual help-seeking behavior in professional rugby football league (RFL) players	Professional rugby players	n=167	NA	Team Sport	Mixed	Mental health, perceived psychological stress, athletic identity, knowledge of player welfare, and actual help-	Quest.	Moderate

Year	Author	Purpose	Sample	Sample size	Gender	Type of Sport	Methodology	Variables	Instruments/Tools	Confidence of evidence
								seeking; barriers to help-seeking		
2019	Kruyt et al.	To explore the perceived psychological demands of the sport and off-the-field needs of professional rugby sevens players	Professional rugby players	n=4 (n=3 male; n=1 female)	Male	Team Sport	Quant	Perceived physical and psychological demands of international sevens and the off-the-field needs of elite players	Semi-structured interviews	Low to moderate
2017	Kuettel et al.	To compare (cross-national similarities and differences) former Swiss, Danish, and Polish elite athletes in terms of processes and outcomes of the transition out of elite sport; to examine and compare the contribution of transitional characteristics on the quality of the transition in these three contexts, building on existing career transition models which consider the transition as a process and where several factors influence the adaptation to a new life or career situation.	Former elite athletes	n=401 (n=269 male; n=132 female)	Mix	Multisport	Quant	Athletic career termination (characteristics of the individual, the career end, and the environment, and their contribution to the quality of the transition in emotional, social, body/health, financial, and vocational areas), transferable skills ((e.g., adaptability, perseverance, performance under pressure, self-awareness, problem-solving, administrative skills) and social support	Quest.	Moderate
2021	Kuettel et al.	To investigate mental well-being and the prevalence of anxiety and depressive symptoms in Danish male and female elite athletes, (b) to identify latent profiles in athletes based on their mental health and ill health, and (c) to examine whether the different profiles vary in selected protective and risk factors concerning mental health	Danish elite athletes	n=612	Mix	Multisport	Quant	Well-being, depression, and anxiety together with potential risk and protective factors (e.g., injuries, stress, sleep, social support, sport environment)	Online Survey Holistic Athlete Mental Health Survey	Moderate to high
2024	Kuettel et al.	To explore elite athletes' perceptions of factors associated with their mental health and thriving based on the combination of holistic developmental and ecological approaches.	Danish international elite athletes	n=7	Mix	Multisport	Qual	Demanding and relentless elite sport context, Personal reactions to elite sport demands, Personal resources and strategies, Supportive and caring environment	Online Interviews	Moderate
2024	Lane	To explore the importance of psychosocial support within professional sporting	Current and retired athletes,	n=17	Mix	Multisport	Qual	Mental Health and Wellbeing; Psychosocial Support; The Athlete Experience; Domains	Semi-structured interviews	Moderate to high

Year	Author	Purpose	Sample	Sample size	Gender	Type of Sport	Methodology	Variables	Instruments/Tools	Confidence of evidence
		organizations and its impact on athlete wellbeing.	Player development managers, athlete wellbeing and education managers, a strength and conditioning head coach					of Interaction; Professional Expectations		
2019	Lebrun et al.	To identify and examine the coping strategies implemented by elite athletes suffering from depression in order to increase our understanding of elite athletes' subjective experience of MHI(s).	Elite athletes (active and former)	n=4 (n=3 male; n=1 female)	Mix	Multisport	Qual	Key events related to their mental health problem(s) and career, experience of depression	Semi-structured interviews	Low to moderate
2025	Liu et al.	To explore the subjective experiences, triggers, and coping strategies of suicidal ideation among Chinese national team elite athletes at various career stages.	Chinese national team elite athletes (international and national level; active, all reached highest competition levels in their sports)	n=10 (5 female, 5 male)	Mix	Multisport	Qual	Triggers and experiences of suicidal ideation, coping strategies, career stage (junior, transition, established), perceived support, cultural factors, social connectedness, stigma	Semi-structured interviews guide; critical realism framework; composite vignettes; reflexive thematic analysis	Moderate
2020	Lundqvist	To describe the use of behavioral activation (Martell et al., 2010) when a former Olympic athlete developed depression after career termination	Olympic athlete	n=1	ND	ND	Quant	Depression; Treatment; Career transition	Clinical interview, self-assessments, psychoeducation, Montgomery Asberg depression rating scale (MADRS-S); Generalized anxiety disorder-7 assessment (GAD-7); Brunnsviken brief quality of life scale (BBQ); DSM-5 self-rated level 1 cross-cutting symptom measure	Low

Year	Author	Purpose	Sample	Sample size	Gender	Type of Sport	Methodology	Variables	Instruments/Tools	Confidence of evidence
									(DSM-5 Self-Rated CCSM)	
2021	Lundqvist et al.	To explore Swedish elite athletes' perceptions of quality of life through IPA	Elite international competitors	n=8	Mix	Multisport	Qual	Intrinsic motivation, stressors, gratitude, goal pursuit, emotional well-being	Semi-structured interviews	Moderate
2020	McLoughlin et al.	To examine how cumulative lifetime stress predicts mental health outcomes and well-being in elite athletes	Elite athletes	n=95 (quantitative); n=6 (qualitative)	Mix	Multisport	Mixed	Cumulative stress, depression, anxiety, well-being	Quest. (Adult STRAIN, PHQ-9, GAD-7, SGWB) and Semi-structured interviews	High
2022	McLoughlin et al.	To examine (a) how lifetime stressor exposure was associated with general physical and mental health complaints among elite athletes and (b) the extent to which these associations were moderated by perfectionism (socially prescribed, self-oriented, or other-oriented).	Elite athletes	n=110 (n=64 female, n=45 male, 1 other)	Mix	Multisport	Quant	Cumulative lifetime stressor exposure, Perfectionism	Survey (online)	Moderate
2023	Miller et al.	To explore the barriers and facilitators to mental health help-seeking behaviors among British elite track and field athletes and provide insights for creating pathways and interventions for mental health support.	Elite athletes	n=9; n=3 male; n= 6 female	Mix	Individual	Qual	Barriers and facilitators to mental health help-seeking	Semi-structured interviews (online)	Moderate
2024	Mooney et al.	To examine how athletic identity, psychological flexibility and valued living impact psychological distress and subjective well-being of retired elite rugby players.	Retired athletes (professional rugby players)	n=77 (n= 64 males, n=13 females)	Mix	Team Sport	Quant	Subjective well-being, psychological distress, psychological flexibility, valued living, athletic identity	Quest.	Moderate
2021	Moreno et al.	To explore the subjective perception of the resources and barriers that employee-athletes have to face in their DC process.	Elite athletes (active and former)	n=18 (n=10 male; n=8 female)	Mix	Multisport	Qual	Athletes' personal experiences in relation to DC, their sources of support, their motivations in relation to work, or their sources of income	Semi-structured interviews	Moderate
2018	Moret et al.	To analyze how social class and sport capital affect education and career transitions among elite Swiss hockey players	Elite hockey players	n=605 quantitative; n=36 qualitative	Male	Team Sport	Mixed	Social class, educational pathway, sport capital, career decisions	Custom survey, NVivo-coded interviews	Moderate

Year	Author	Purpose	Sample	Sample size	Gender	Type of Sport	Methodology	Variables	Instruments/Tools	Confidence of evidence
2024	Murdoch and Hong	To explore the experiences of British elite swimmers in developing life skills during and throughout their athletic careers, examining the factors that influence their perspectives on this skill development.	Elite athletes (active and former)	n=6 (n=6 male; n=6 female)	Mix	Individual	Qual	Participants' athletic careers and their life skills throughout their athletic careers, including following key areas: (a) beginning of swimming career; (b) development and learning throughout their swimming career; (c) life skills development; and (d) reflection on career impact and life post-swimming.	Semi-structured interviews	Moderate
2016	Newman et al.	To explore depressive experiences in elite athletes and how these relate to sport performance and post-career identity	Elite athletes	n=12	Mix	Multisport	Qual	Depression, identity, performance, emotional impact of retirement	Autobiographical content analysis	Moderate
2023	Oforeh et al.	To highlight the adverse consequences of poorly managed stress in an elite athlete	Elite professional basketball player	n=1	Male	Team Sport	Quant	Stress, psychiatric illness, affective disorders, career injuries	Clinical psychiatric evaluation	Low to moderate
2023	Oguro et al.	To explore mental health help-seeking preferences and behavior among elite male rugby players	Elite male rugby players	n=219 (only 20% had experience in national team)	Male	Team Sport	Quant	Help-seeking preference, stigma, access to support	Survey (Self-reported online)	Low to moderate
2021	Ojio et al.	To examine the prevalence of mental health problems and suicidal ideation and its risk factors in Japan Rugby Top League players.	Japan Rugby Top League players	n=251	Male	Team Sport	Quant	Japanese version of the 6-item Kessler-6. Suicidal ideation was assessed using the Baron Depression Screener for Athletes.	Quest.	Moderate
2021	Oltmans	To establish the 12-month incidence and comorbidity of symptoms of mental health disorders (distress, anxiety/depression, sleep disturbance, alcohol misuse, disordered eating)	Elite athletes	n=193	Mix	Multisport	Qual	Involuntary retirement, number of recent life events, and career dissatisfaction were measured, Symptoms of distress, Anxiety/depression, sleep disturbance, alcohol misuse, and disordered eating were measured at baseline	Quest.	Moderate to high
2020	Oulevey et al.	To further advance research in this area, with a specific focus the developmental experiences related to the sport career transition process in Japan.	Olympic medalist	n=1	Female	Individual	Qual	1) Starting synchro; 2) Deepening commitment; 3) Competitive characteristics; 4) Sporting values; 5) Retirement difficulties; 6) Current challenges; 7) New values; 8) Transferable skills; and 9)	Semi-structured interviews	Low to moderate

Year	Author	Purpose	Sample	Sample size	Gender	Type of Sport	Methodology	Variables	Instruments/Tools	Confidence of evidence
								Advice for the next generation.		
2018	Papathomas	To examine body image perceptions and experiences among retired female athletes from aesthetic sports, focusing on changes after retirement.	Retired elite female collegiate athletes (gymnastics, swimming; aesthetic, weight-sensitive sports)	n=218 (Gymnastics (66%), swimming (34%))	Female	Multisport	Mixed	Weight perception, weight satisfaction, coping with body change, body composition, years since retirement, internalized ideals (athletic, feminine)	Online survey: demographic questions, closed-ended (weight, satisfaction), open-ended (perceived changes, coping strategies)	Moderate to high
2025	Papathomas et al.	To explore the experiences of retired elite male and female athletes, persistently exposed to unique weight-based pressures and intense body scrutiny, in relation to their body image and satisfaction during retirement	Former elite athletes	n=31 (n= 9 male; n= 22 female)	Mix	Multisport	Qual	body image during retirement from sport, retirement decision, perceived body changes, the meaning of these changes, and associated coping strategies	Semi-structured interviews (online)	Moderate to high
2023	Parrott	To examine how elite athletes' testimonials (Players' Tribune) challenge and elucidate myths about mental health in sport, focusing on stigma and disclosure.	Elite athletes (current and retired; Olympic, NBA, NFL, WNBA, NHL, etc.)	n=37 first-person essays	Mix	Multisport	Qual	Mental health myths, stigma, identity, success, strength, transition, help-seeking, isolation, retirement	Braun & Clarke's thematic analysis; qualitative coding	High
2022	Perry et al.	To explore the prevalence and factors associated with depression, anxiety, and eating disorder symptoms in elite female footballers in England and examine help-seeking intentions.	Elite female footballers	n=115	Female	Team Sport	Qual	Depression (PHQ-9), anxiety (GAD-7), eating disorder symptoms (BEDA-Q), help-seeking intentions (GHSQ), starting status, student-athlete status, paid contract, injury, need for psychological support	Quest. (QPHQ-9, GAD-7, BEDA-Q, GHSQ) and demographic survey	High
2021	Poucher et al.	To evaluate the prevalence of symptoms of mental health disorders, specifically depression, anxiety, and eating disorders (EDs), among elite Canadian athletes, and identified the relationship between components of the stress process model (stress,	National team athletes	n=186	Mix	Team Sport	Quant	Symptoms of mental health disorders, depression, anxiety, and eating disorders stress process model (stress, social support, coping, and self-esteem)	Survey	Moderate

Year	Author	Purpose	Sample	Sample size	Gender	Type of Sport	Methodology	Variables	Instruments/Tools	Confidence of evidence
		social support, coping, and self-esteem) and athletes' symptoms of mental disorders.								
2023	Poucher, et al	To investigate how elite sport training environments in Canada impact the mental health of the athletes who train within these spaces. This research was guided by theoretical understandings of organizational stressors and factors that are associated with athletes' mental health, which provided an initial starting point for investigating the links between these connections. Specifically, we were interested in answering the following questions: 1) How does involvement with elite sport organizations impact athletes' perceptions of mental health? 2) What are the features of Canadian elite sport environment that support or detract from athlete mental health?	Olympic and Paralympic Canada Team	n=32 (15 female, 9 Olympic and 6 paralympic, and 17 male (12 Olympic and 5 Paralympic)	Mix	Multisport	Qual	personal experiences with mental health, and their perceptions of the organizational factors that supported or detracted from their mental health.	Survey	Moderate
2024	Prior et al.	To explore performance directors' understanding and management of athlete mental health	Elite athletes (Olympic, professional, semiprofessional) managed by performance directors	n=11 performance directors	Mix	Multisport	Qual	Mental health perception, stigma, responsibility narratives	Semi-structured interviews	Moderate
2025	Prior et al.	To explore sport psychologists' perceptions of their role in relation to athlete mental health and illness and their experiences of offering mental health support.	Sport psychologists of elite athletes	n=12	Mix	Multisport	Qual	A lack of consensus on remit and role; organization-practitioner misaligned expectations; and the emotional toll of supporting athlete mental health.	Semi-structured interviews	Moderate
2020	Raabe et al.	To qualitatively explore the role of individuals' basic psychological needs during the release from professional baseball and	Former Minor League Baseball players	n=12	Male	Team Sport	Qual	Basic psychological needs, self-determination theory	Semi-structured interviews	Moderate

Year	Author	Purpose	Sample	Sample size	Gender	Type of Sport	Methodology	Variables	Instruments/Tools	Confidence of evidence
		throughout their subsequent transition to a new career								
2021	Ramos et al.	To examine the dual career and entrepreneurial experiences of professional football players and their influence on the career transition process to entrepreneurship or employment.	Professional football players	n=8809	Male	Team Sport	Quant	Return (dummy) If the former football player pursues a second career as employee after retirement; Entrepreneur (dummy) If the former football player pursues a second career as entrepreneur after retirement; Education level (dummy) Number of years completed at school; Portuguese (dummy) If the individual is Portuguese; Retirement age (years) Individual's age at the time of retirement from football; Gap (years) Time period between retirement and second career; Number of clubs Number of clubs in which the individual has played; Player career length (years) Number of years the individual was a player; Additional job once If the player had an additional job during anytime of the football career; Additional job last year If the player had an additional job during the last year of the football career; Last salary (euros) The value of the salary received in the last year of the professional player career; Total salary (euros) The total value accumulated in salaries in all years of professional playing career in Portugal; Last league Highest league The football league in which the individual played during the last year of his	Survey	Moderate

Year	Author	Purpose	Sample	Sample size	Gender	Type of Sport	Methodology	Variables	Instruments/Tools	Confidence of evidence
								football career; The highest football league in which the individual played during his career; Entrepreneur once(dummy) If the individual had gained entrepreneurial experience during the player career		
2022	Robnik et al.	To examine the impact of factors directly contributing to the quality of the post-sport career transition in Slovenian elite and Olympic athletes and the social class position and employment of these athletes after the termination of their sports career	Elite athletes (active and former)	n=168 (n= 102 male; n= 66 female)	Mix	Multisport	Quant	Social-class position of former elite athletes 5 years after their sports career termination (CLASS); educational level; DC support programs (including (1) sports scholarships, (2) tutorship program, (3) cofinancing of tuition, (4) Distance Study Program, (5) human resources development in sports project, (6) Professional Athlete Career Program, and (7) employment of athletes in public administration); best results/sports achievements; reasons for sports career termination and the decision to retire from elite sports	Quest.	Moderate
2024	Rohlfs et al.	To assess mood profiles and risk factors for mental ill-health among elite and youth athletes	Elite and youth athletes (club level)	n=898	Mix	Multisport	Quant	Mood states, mental health risk	Quest. (Brazil Mood Scale (BRAMS))	Moderate
2016	Ronkainen et al.	To explore gendered experiences of the mastery stage in endurance runners' athletic careers in terms of (a) key themes in this period of life, (b) retirement decision-making and (c) changes in athletic and runner identities	National athletes	n=19	Mix	Multisport	Qual	Characteristics/Behaviours; Sport Psychology; Identity; Athletic retirement; Culture	Individual interviews	High
2023	Röthlin et al.	To explore current mental health provisions in Switzerland.	Sport organizations and athlete	n=31	Mix	Multisport	Quant	Mental health plan, mental health care, athlete support system and high-risk events	Survey	Low to moderate

Year	Author	Purpose	Sample	Sample size	Gender	Type of Sport	Methodology	Variables	Instruments/Tools	Confidence of evidence
2018	Ryan	To examine the experiences of selected New Zealand carded athletes and explore how expectations related to the normative sport ethic, and process of performance-based funding, encourage the prioritization of a sport-only identity, and explores ways in which athletes challenge the pervasiveness of this identity.	Elite athletes (active and former)	n=17 (n=7 male; n=10 female)	Mix	Multisport	Qual	Gather a holistic perspective of life as a carded athlete and focused on athlete's perceptions of how being part of the carding system has impacted their identity development both in, and outside, of the sport environment	Semi-structured interviews	Moderate
2023	Sauvé et al.	To examine the perspectives of Olympic coaches and national performance directors regarding factors they felt influence athlete well-being (both positively and negatively), and to explore their perceived roles in contributing to the well-being of their athletes	International coaches and directors of Olympic athletes	Coaches (n = 12); National performance directors (n = 12)	Mix	Multisport	Qual	Relentless pursuit of results; lack of opportunities outside of sport; focus on results as harmful; influence of the coach; coach as the relationship builder; coach as the gatekeeper of well-being; Financial uncertainty of the program/for the coach	Semi-structured interviews	Moderate
2022	Schmid et al.	To examine patterns of sports-related and socio-economic resources at the time of athletic retirement and their relation to the subsequent vocational career	Olympic Athletes	n=341	Mix	Multisport	Qual	The athletic, educational, and vocational career (e.g. Major achievements in sports, age at the time of athletic retirement, duration of sports career; type of education, highest educational qualification and duration of education; job title and position; education and profession of parent)	Quest.	High
2023	Schmid, et al.	To examine the complex associations between athletic retirement and self-esteem among former elite athletes.	Swiss Olympic athletes	n=290	Mix	Multisport	Quant	Sporting career success; Sporting career satisfaction; Athletic identity; Self-esteem; Transition characteristics; Short- and medium-term adjustment to, and consequences of, career termination;	Quest.	High
2024	Schmid, et al.	To assess the development of athletic identity over several years spanning from the athletic to the post-athletic career, and to examine to what extent gender,	Former elite athletes	n=290; n=195 male; n=95 female	Mix	Multisport	Quant	Sport-specific information (type of sport, start/end of career, achievements), athletic identity, athletic career age, training effort, status (semi-	Quest.	High

Year	Author	Purpose	Sample	Sample size	Gender	Type of Sport	Methodology	Variables	Instruments/Tools	Confidence of evidence
		age, career age, sporting success, voluntariness of career termination, post career involvement in sport, and life roles, explain interindividual differences in levels and trajectories of athletic identity.						professional or professional), objective and subjective sporting success, voluntariness of career termination. involvement in sports after career termination, salience of different life roles (i.e., sport, education, occupation, leisure, relationships, family etc.) and self-complexity		
2021	Silver	To examine how high-performance athletes' perceptions about aging influenced their adaptation to athletic retirement	Olympic Athletes	n=24	Mix	Multisport	Qual	Perceptions About Aging Influenced Exercise Participation in Retirement, Perceptions About Aging Motivated Participants to Engage in Civic-Minded Postretirement Activities, Lack of Perceptions about Aging, Loss and Sense of Purpose	Quest.	Moderate
2020	Smismans et al.	To develop and initially validate an instrument that measures athletes' competencies required to optimize their employability across different phases of athletic retirement.	Elite athletes (active and former)	n=954 (n= 515 male; n= 439 female)	Mix	Multisport	Quant	Career & Lifestyle Management (CLM), Career Communication (CCO), Career Resilience (CRE), and Career Engagement & Flexibility (CEF)	Quest.	Moderate
2025	Steinfeldt et al.	To assess the effect of income shocks on elite athletes' life satisfaction and career-ending thoughts during COVID-19	German National/Olympic elite athletes	n=1652	Mix	Multisport	Quant	Income shock, life satisfaction, career-ending thoughts	Quest.	Moderate
2015	Stronach et al.	To investigate the retirement experiences of Indigenous Australian sportsmen	Indigenous elite athletes	n=30	Male	Multisport	Qual	Engaging indigeneity Personal identity; Indigenous heritage and culture; 'Natural athletes'; Family cohesion and connectedness; Athletic retirement Exiting sport; Nowhere Land; New beginnings; Moving on Athlete career and education programs; Career choices and stereotyping; Support during	open-ended and conversational interview	Moderate

Year	Author	Purpose	Sample	Sample size	Gender	Type of Sport	Methodology	Variables	Instruments/Tools	Confidence of evidence
								and after sport; Indigenous context of athletic retirement		
2024	Teixeira et al.	To assess Quality of Life among Portuguese former players according to career duration, career end period, competitive level, tactical-positional status, international status, academic qualifications, serious injuries in career, and current professional football connection	National team athletes	n=84	Male	Team Sport	Quant	Quality of life: Physical Pain and Discomfort; Energy and Fatigue; Sleep and Rest; Mobility; Activities of Daily Living; Dependence on Medication or Treatments; Work Capacity; Psychological Positive feelings; Thoughts, feelings, memory and concentration; Self-esteem; Body image and appearance; Negative feelings; Spirituality/Religion/Beliefs; Social relationships; Personal relationships; Social support; Sexual activity; Environment; Physical security; Home environment; Economic Resources; Health and social care; Opportunities to acquire new information and skills; Participation or opportunities for recreation and leisure; Physical environment (pollution/noise/traffic/climate); Transport	Quest. (WHOQOL-BREF)	Low to moderate
2024	Thornton et al.	To investigate retired elite female rugby players' health outcomes (and their relationships) in five key areas (musculoskeletal, cognitive, mental, reproductive/endocrinological and cardiovascular) and how those compare with the general population.	Elite athletes (at least five international matches/national team)	National performance directors (n = 12)	Female	Team Sport	Quant	Injury history (number, site), persistent pain, HOOS, KOOS, FAOS, Oswestry Disability Index, Brief Pain Inventory, Concussion diagnosis, CNS Vital Signs cognitive testing (memory, attention, psychomotor speed, etc.). Psychological distress (K10), anxiety (GAD-7), depression (PHQ-9), alcohol/drug abuse (AUDIT-C, CAGE-AID), eating disorders (EDE-Q), harassment/abuse experiences.	Online Quest. (179-item online questionnaire and neurocognitive assessment)	Moderate

Year	Author	Purpose	Sample	Sample size	Gender	Type of Sport	Methodology	Variables	Instruments/Tools	Confidence of evidence
								Amenorrhea, age at menarche/menopause, fertility/pregnancy history, pregnancy diagnoses (gestational diabetes, preeclampsia). Cardiovascular diagnoses (hypertension, diabetes, cholesterol), sleep quality (PSQI), post-career physical activity levels.		
2015	Torregrosa et al.	To evaluate the influence that following a dual career or being exclusively focused in sport can have in the retirement process of Olympians with excellent sporting achievements.	Former elite athletes	n=15 (n=10 male; n=5 female)	Mix	Multisport	Qual	Review of the sporting career until retirement from competitive sport, analysis of the retirement process (planned or unplanned), review of the transition period looking into its length and assessing how the athlete coped at the sporting, personal, relational and vocational levels, the institutional support received or not during the period of transition, personal view of retirement from elite sport.	Semi-structured interviews	Moderate
2022	Trudel et al.	To present and analyze a tailored, workplace-based learning journey designed to support the transition of an elite athlete (Dan) into a certified high-performance coach in Canada.	Former high-performance (national/international) volleyball player transitioning to HP coach	n=1	Male	Team Sport	Qual	Career transition, learning process, coach education, mentoring, individualized curriculum, certification requirements, reflective practice	Coaching portfolio, learning trajectory mapping, coaching conversation sessions (45 meetings), thematic documentation	Low to moderate
2015	Tshube et al.	To get an in-depth account of the role of dual careers on elite athletes' post-sport career transition and to examine these issues cross-culturally between South Africa, Botswana, Namibia and Zimbabwe	Elite retired athletes	n=17	Mix	Multisport	Qual	Dual-career challenges in Botswana, Namibia, South Africa and Zimbabwe; Dual career facilitates retirement transition;	Online survey, semi-structured interviews and focus groups	Moderate

Year	Author	Purpose	Sample	Sample size	Gender	Type of Sport	Methodology	Variables	Instruments/Tools	Confidence of evidence
2018	Tshube et al.	To examine career development of elite athletes in Southern Africa, their psychosocial development across the phases of elite lifespan development, as well as their career transition experiences	Retired Olympic athletes	N=16	Mix	Multisport	Qual	Athletes' entourage profiles, Athletic Career Stages	Semi-structured interviews	Low
2021	Van Patten et al.	To explore predictors and correlates of perceived cognitive decline among retired professional rugby league players	Retired elite rugby league players	n=133	Male	Team Sport	Quant	Perceived cognitive decline, depression, concussion history, resilience	Quest. (IQCODE-Self, DASS-21, CD-RISC, neuropsychological battery)	Moderate
2014	Vilanova et al.	To examine how Spanish Olympians experience the transition to a second career; (2) to identify the strategies which, based on this experience, they may or may not implement in order to prepare for it; and (3) to determine the factors that influence the behaviour observed	Olympians	n=26 retired from 4-12 years	Mix	Multisport	Qual	Awareness of time (always, at some point, never); Actions taken (academic training, working before ending their sporting career, saving money, taking advantage of their sporting capital and voluntary retirement); Significant others (family, coach, club, partner, siblings); Subjective perception of their actual job (feeling lucky, satisfaction, resignation).	Semi-structured interviews	Moderate to high
2017	Vilanova et al.	To establish a typology of job market entry strategies among Olympic athletes.	Former Olympic athletes	n=94	Mix	Multisport	Quant	Parallel life strategists; Freelance strategists; Lifetime athletes; non-strategists: it's a job	Survey and semi-structured interviews	Moderate
2023	Willson et al.	To explore the relationships between athletes' experiences of maltreatment and mental health indicators. Canadian National Team athletes completed an online, anonymous survey that assessed reported experiences of maltreatment (psychological, physical, sexual harm and neglect), and mental health indicators of well-being, eating disorders and self-harming behaviours.	Canadian national team athletes	n=995	Mix	Multisport	Quant	Gender Disability Race; Sexual Orientation; Indigenous Neglect Psychological Harm; Physical Harm; Sexual Harm; Self Harm; Eating Disorder; Wellbeing	Survey	High

Year	Author	Purpose	Sample	Sample size	Gender	Type of Sport	Methodology	Variables	Instruments/Tools	Confidence of evidence
2025	Willson et al.	To examine the effects of psychological abuse on athlete satisfaction and mental health indicators of eating disorders and self-harm	Elite athletes (active and former)	n=794 (n=295 male; n=496 female; n=3 not declared)	Mix	Multisport	Quant	Psychological abuse, athletes' satisfaction, eating disorder indicators, self-harm indicators	Quest.	Moderate to high
2021	Yongrui et al	To analyze five factors, including gender, psychological capital, human capital, individual social influence of winning sport event and 'cross-border distance' using qualitative comparative analysis (QCA).	Chinese retired Olympic champions	n=80	Mix	Multisport	Qual	Gender, psychological capital, human capital, sport event influence, cross-border distance, cross-border success, cross-border failure	qualitative comparative analysis (QCA)	Moderate to high
2024	Young et al	To evaluate the effectiveness and perceived value of "MORE THAN SPORT" (MTS), a digital social-identity intervention to support elite athletes' identity management during transition out of elite sport.	Former or retiring elite athletes from 15 sports (individual & team) multinational Sample (UK, Australia, Belgium)	n=25 (11 female, 14 male)	Mix	Multisport	Qual	Experiences with transition, identity loss, identity management, social identity mapping, perceptions of program content and delivery, perceived support needs	Online MTS intervention, Semi-structured interviews, social identity mapping, Qualitative coding (NVivo)	Moderate to high
2023	Yufeng et al.	To advance athlete career scholarship using meta-transitions within the Chinese Whole Nation System (CWNS). The intentions are to: (a) provide in-depth understandings of Chinese elite athletes' careers through shared dialog, (b) transform knowledge and support prospective athletes, and (c) contribute to the advancement of the CWNS by exploring its impact on elite athletes' career development and transitions.	Elite athletes – first level (pre-national team level)	n=3	Mix	Team Sport	Qual	Meta-transitions, cultural context, coping strategies, career development	Group reflective practice, polyphonic reflective tale	Moderate
2017	Zurc	To investigate the opinions of female gymnasts on their sports careers and top achievements in artistic gymnastics.	Elite Artistic Gymnastics Athletes	n= 26 active Slovenian female gymnasts; n=11 retired Slovenian	Female	Individual	Qual	Elite child athlete, ethics, wellbeing, reasons for starting to train gymnastics, parents' role, raining characteristics, competitions, balancing school and gymnastics, adverse	In-depth retrospective Semi-structured interviews	Moderate

Year	Author	Purpose	Sample	Sample size	Gender	Type of Sport	Methodology	Variables	Instruments/Tools	Confidence of evidence
				female gymnasts; n=4 coaches				events, gymnastics as a way of life, development of talent, identification of talent, the process of making an elite gymnast/development of talent, legacy after career conclusion, doing it again, making changes to past career		
