# Supplementary material for: Sustainable Career Transitions and Mental Health Support in Elite Sport: A Systematic Review of Evidence and Practices
Source: Sports (Basel). 2025 Dec 5;13(12):438. doi: 10.3390/sports13120438 (PMC12813639; doi:10.3390/sports13120438)
Supplement: Supplementary file 1 [file sports-13-00438-s001.zip › 4_Supplementary File S4_Mixed Methods Appraisal Tool (MMAT) checklist items.pdf]

Supplementary material S4 - Mixed Methods Appraisal Tool (MMAT) checklist items.

	**Methodological quality criteria**
**1. Screening questions (for all types)**	**1.1.** Are there clear research questions?<br>**1.2.** Do the collected data allow to address the research questions?
**2. Qualitative studies**	**2.1.** Is the qualitative approach appropriate to answer the research question?<br>**2.2.** Are the qualitative data collection methods adequate to address the research question?<br>**2.3.** Are the findings adequately derived from the data?<br>**2.4.** Is the interpretation of results sufficiently substantiated by data?<br>**2.5.** Is there coherence between qualitative data sources, collection, analysis and interpretation?
**3. Quantitative descriptive studies**	**3.1.** Is the sampling strategy relevant to address the research question?<br>**3.2.** Is the sample representative of the target population?<br>**3.3.** Are the measurements appropriate?<br>**3.4.** Is the risk of nonresponse bias low?<br>**3.5.** Is the statistical analysis appropriate to answer the research question?
**4. Mixed method studies**	**4.1.** Is there an adequate rationale for using a mixed methods design to address the research question?<br>**4.2.** Are the different components of the study effectively integrated to answer the research question?<br>**4.3.** Are the results adequately brought together into overall interpretations?<br>**4.4.** Are divergences and inconsistencies between quantitative and qualitative results adequately addressed?<br>**4.5.** Do the different components of the study adhere to the quality criteria of each tradition of the methods involved?

Summary of the Mixed Methods Appraisal Tool item-level judjements across all included studies.

Mixed Methods Appraisal Tool item	Yes (n, %)	No (n, %)	Can't tell (n, %)
Q1	116 (99%)	1 (1%)	0 (0%)
Q2	117 (100%)	0 (0%)	0 (0%)
Q3	110 (94%)	0 (0%)	7 (6%)
Q4	97 (83%)	0 (0%)	20 (17%)
Q5	111 (95%)	0 (0%)	6 (5%)
Q6	72 (62%)	5 (4%)	40 (34%)
Q7	105 (90%)	1 (1%)	11 (9%)

Note: Counts (n) and percentages (%) of "Yes", "No", and "Cant'tell" responses for each MMAT checklist item (Q1-Q7). Total refers to the number of studies evaluated per item.

Distribution of Mixed Methods Appraisal Tool checklist across included studies (n=117).

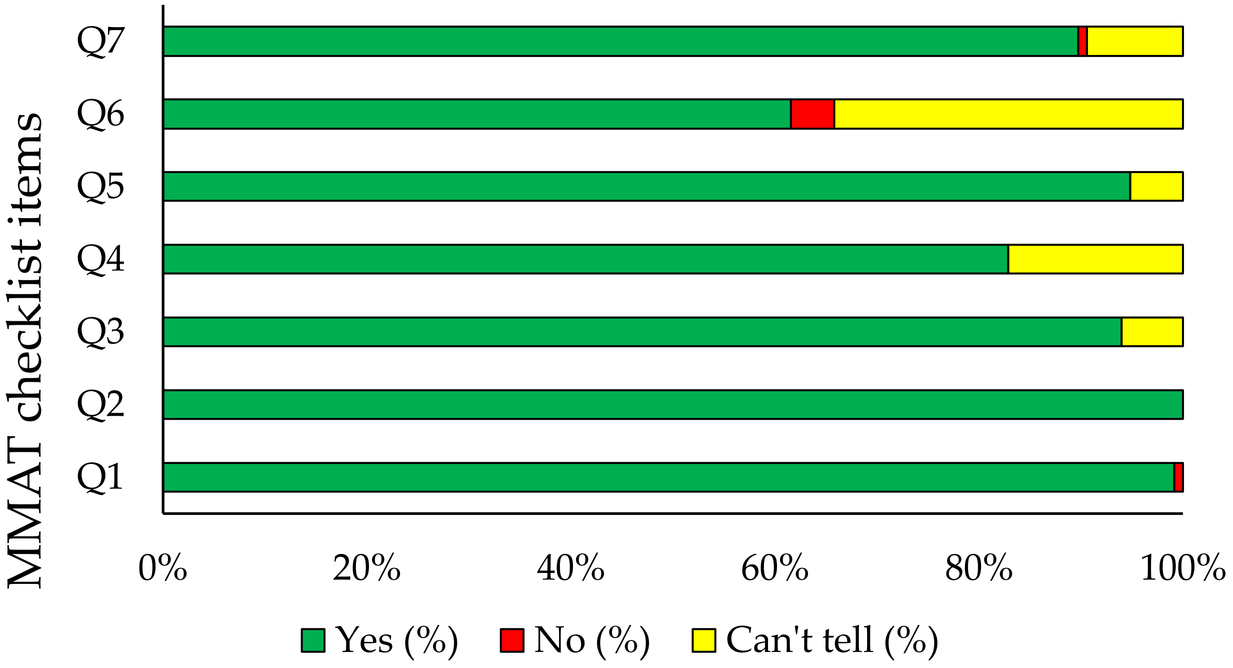

Evaluation of the included studies according to MMAT screening questions

		Transversal questions		Specific questions based on the study design				
Year	Methodology	Question 1	Question 2	Question 3	Question 4	Question 5	Question 6	Question 7
2015	Qualitative	yes	yes	yes	CT	yes	no	yes
2015	Qualitative	yes	yes	yes	yes	yes	yes	CT
2015	Qualitative	yes	yes	yes	yes	yes	yes	CT
2015	Quantitative	yes	yes	CT	yes	yes	CT	yes
2015	Qualitative	yes	yes	yes	CT	yes	CT	yes
2015	Qualitative	yes	yes	yes	yes	yes	yes	yes
2016	Qualitative	yes	yes	yes	yes	yes	yes	yes
2016	Qualitative	yes	yes	yes	yes	yes	yes	yes
2016	Quantitative	yes	yes	CT	yes	yes	CT	yes
2016	Qualitative	yes	yes	yes	yes	yes	yes	yes
2017	Qualitative	yes	yes	yes	yes	yes	yes	CT
2017	Qualitative	yes	yes	yes	yes	yes	yes	yes
2017	Qualitative	yes	yes	CT	yes	yes	CT	yes
2017	Quantitative	yes	yes	CT	yes	yes	CT	yes
2017	Quantitative	yes	yes	yes	CT	yes	CT	yes
2017	Qualitative	yes	yes	yes	yes	yes	yes	yes
2017	Quantitative	yes	yes	yes	CT	yes	no	yes
2018	Qualitative	yes	yes	yes	yes	yes	yes	yes
2018	Qualitative	yes	yes	yes	CT	yes	CT	CT
2018	Qualitative	yes	yes	yes	yes	yes	yes	yes
2018	Qualitative	yes	yes	yes	yes	yes	yes	yes
2018	Mixed-Methods	yes	yes	yes	CT	yes	CT	yes
2018	Qualitative	yes	yes	yes	yes	yes	yes	yes
2018	Mixed-Methods	yes	yes	yes	yes	yes	yes	yes
2018	Quantitative	yes	yes	yes	yes	yes	CT	yes
2018	Qualitative	yes	yes	yes	yes	yes	CT	yes
2018	Mixed-Methods	yes	yes	yes	yes	yes	yes	yes
2018	Qualitative	yes	yes	yes	yes	yes	yes	yes
2018	Qualitative	yes	yes	yes	yes	yes	yes	yes
2019	Qualitative	yes	yes	yes	yes	yes	yes	yes
2019	Quantitative	yes	yes	yes	CT	yes	CT	yes
2019	Qualitative	yes	yes	yes	yes	CT	CT	yes
2019	Quantitative	yes	yes	yes	yes	CT	CT	yes
2019	Quantitative	yes	yes	yes	yes	yes	CT	yes
2020	Qualitative	yes	yes	yes	yes	yes	yes	CT
2020	Quantitative	yes	yes	yes	yes	yes	CT	yes
2020	Quantitative	yes	yes	yes	yes	yes	CT	CT
2020	Qualitative	yes	yes	yes	yes	yes	yes	yes
2020	Quantitative	yes	yes	yes	yes	CT	CT	yes
2020	Mixed-Methods	yes	yes	yes	yes	yes	yes	yes
2020	Quantitative	yes	yes	CT	yes	yes	CT	yes
2020	Quantitative	yes	yes	yes	yes	yes	yes	yes
2020	Mixed-Methods	yes	yes	yes	CT	CT	yes	yes
2020	Quantitative	yes	yes	yes	CT	yes	yes	yes
2020	Quantitative	yes	yes	yes	CT	yes	CT	yes
2020	Qualitative	yes	yes	yes	yes	yes	yes	yes
2020	Qualitative	yes	yes	yes	yes	yes	yes	yes
2021	Quantitative	yes	yes	yes	yes	yes	CT	yes

2021	Quantitative	yes	yes	yes	yes	yes	CT	yes
2021	Quantitative	yes	yes	CT	yes	yes	yes	yes
2021	Quantitative	yes	yes	CT	yes	yes	CT	yes
2021	Qualitative	yes	yes	yes	yes	yes	yes	yes
2021	Mixed-Methods	yes	yes	yes	yes	yes	yes	yes
2021	Qualitative	yes	yes	yes	yes	yes	CT	yes
2021	Qualitative	yes	yes	yes	yes	yes	CT	yes
2021	Qualitative	yes	yes	yes	yes	yes	yes	yes
2021	Qualitative	yes	yes	yes	yes	yes	yes	yes
2021	Quantitative	yes	yes	yes	CT	yes	CT	yes
2021	Qualitative	yes	yes	yes	yes	yes	yes	yes
2021	Qualitative	yes	yes	yes	yes	yes	yes	yes
2021	Qualitative	yes	yes	yes	yes	yes	yes	yes
2021	Qualitative	yes	yes	yes	yes	yes	yes	yes
2022	Qualitative	yes	yes	yes	yes	yes	yes	yes
2022	Qualitative	yes	yes	yes	yes	yes	yes	yes
2022	Quantitative	yes	yes	yes	yes	yes	yes	yes
2022	Quantitative	yes	yes	yes	yes	yes	no	yes
2022	Qualitative	yes	yes	yes	yes	yes	yes	yes
2022	Qualitative	yes	yes	yes	yes	yes	yes	yes
2022	Qualitative	yes	yes	yes	yes	yes	yes	yes
2022	Quantitative	yes	yes	yes	yes	yes	CT	yes
2022	Quantitative	yes	yes	yes	CT	yes	CT	yes
2022	Qualitative	no	yes	yes	yes	yes	yes	yes
2022	Qualitative	yes	yes	yes	yes	yes	yes	yes
2022	Qualitative	yes	yes	yes	yes	yes	yes	yes
2022	Quantitative	yes	yes	yes	yes	yes	CT	yes
2022	Quantitative	yes	yes	yes	CT	yes	CT	yes
2023	Qualitative	yes	yes	yes	yes	yes	yes	yes
2023	Qualitative	yes	yes	yes	yes	yes	yes	CT
2023	Qualitative	yes	yes	yes	yes	yes	yes	CT
2023	Quantitative	yes	yes	yes	yes	yes	yes	yes
2023	Qualitative	yes	yes	yes	yes	yes	yes	yes
2023	Quantitative	yes	yes	yes	yes	yes	yes	yes
2023	Qualitative	yes	yes	yes	yes	yes	yes	yes
2023	Qualitative	yes	yes	yes	yes	yes	yes	yes
2023	Quantitative	yes	yes	yes	yes	CT	no	CT
2023	Qualitative	yes	yes	yes	yes	yes	yes	yes
2023	Quantitative	yes	yes	yes	yes	yes	yes	CT
2023	Quantitative	yes	yes	yes	CT	yes	CT	yes
2023	Qualitative	yes	yes	yes	yes	yes	yes	yes
2023	Qualitative	yes	yes	yes	yes	yes	yes	yes
2023	Quantitative	yes	yes	yes	yes	yes	yes	no
2023	Quantitative	yes	yes	yes	CT	yes	no	yes
2024	Mixed-Methods	yes	yes	yes	yes	yes	yes	CT
2024	Qualitative	yes	yes	yes	yes	yes	yes	yes
2024	Quantitative	yes	yes	yes	yes	yes	yes	yes
2024	Quantitative	yes	yes	yes	yes	yes	CT	yes
2024	Quantitative	yes	yes	yes	CT	yes	CT	yes
2024	Qualitative	yes	yes	yes	CT	yes	CT	yes
2024	Qualitative	yes	yes	yes	yes	yes	yes	yes
2024	Qualitative	yes	yes	yes	yes	yes	yes	yes

2024	Quantitative	yes	yes	yes	yes	yes	yes	yes
2024	Quantitative	yes	yes	yes	yes	yes	CT	yes
2024	Qualitative	yes	yes	yes	yes	yes	yes	yes
2024	Qualitative	yes	yes	yes	yes	yes	yes	yes
2024	Quantitative	yes	yes	yes	CT	yes	CT	yes
2024	Quantitative	yes	yes	yes	CT	yes	CT	yes
2024	Qualitative	yes	yes	yes	yes	yes	yes	yes
2024	Qualitative	yes	yes	yes	yes	yes	yes	yes
2024	Qualitative	yes	yes	yes	yes	yes	yes	yes
2024	Quantitative	yes	yes	yes	CT	yes	CT	yes
2025	Qualitative	yes	yes	yes	yes	yes	yes	yes
2025	Mixed-Methods	yes	yes	yes	yes	yes	yes	yes
2025	Qualitative	yes	yes	yes	yes	yes	yes	yes
2025	Quantitative	yes	yes	yes	yes	CT	CT	yes
2025	Qualitative	yes	yes	yes	yes	yes	CT	yes
2025	Quantitative	yes	yes	yes	yes	yes	CT	yes
2025	Qualitative	yes	yes	yes	yes	yes	yes	yes

Note: Yes: criterion met; No: criterion not met; CT= Can’t tell.
